# Fluorinated Carnitine Derivatives as Tools to Visualise Carnitine Transport and Metabolism

**DOI:** 10.1002/advs.202514668

**Published:** 2025-11-07

**Authors:** Richard S. Edwards, Ella‐May Hards, Sofia N. dos Santos, Hannah E. Greenwood, Madeleine E. George, Andrea Emanuelli, Muhammet Tanc, Thomas R. Eykyn, Timothy H. Witney

**Affiliations:** ^1^ School of Biomedical Engineering & Imaging Sciences King's College London London SE1 7EH UK; ^2^ British Heart Foundation Centre of Research Excellence King's College London London SE1 7EH UK; ^3^ Department of Chemistry King's College London London SE1 1DB UK

**Keywords:** cancer, carnitine, metabolism, molecular imaging, PET

## Abstract

Carnitine and its acyl derivatives are essential for the transport of fatty acids from the cytosol into the mitochondrial matrix for β‐oxidation, which supplies the cell with energy. Altered transport and metabolism of carnitine are associated with multiple diseases and disorders, including heart disease, insulin resistance, and cancer. Fluorinated carnitine derivatives have the potential to measure aberrant carnitine metabolism in these disorders using ^19^F‐NMR and mass spectrometry. Furthermore, by radiolabeling carnitines with fluorine‐18, altered carnitine utilisation may be visualised in vivo using positron emission tomography (PET) imaging. Here, the design and synthesis of a fluorinated carnitine derivative, fluoromethylcarnitine (FMC), and its radiolabelled equivalent, [^18^F]fluoromethylcarnitine ([^18^F]FMC), are described, and their ability to quantitatively measure carnitine transport and downstream metabolism in a variety of settings are shown, from simple cell models to living subjects. Finally, [^18^F]FMC PET is used to visualise elevated carnitine utilisation in a xenograft model of non‐small cell lung cancer.

## Graphical abstract

Fluorinated carnitines, fluoromethyl carnitine (FMC) and [^18^F]fluoromethyl carnitine ([^18^F]FMC), are synthesised and established as powerful probes to interrogate carnitine biology. The multimodal detection facilitated by fluorine labelling, including ^19^F NMR, mass spectrometry, and positron emission tomography imaging, allowed for visualisation of carnitine utilisation in a range of systems, from simple cells to living subjects.

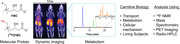

## 1 Introduction

**Figure 1:**
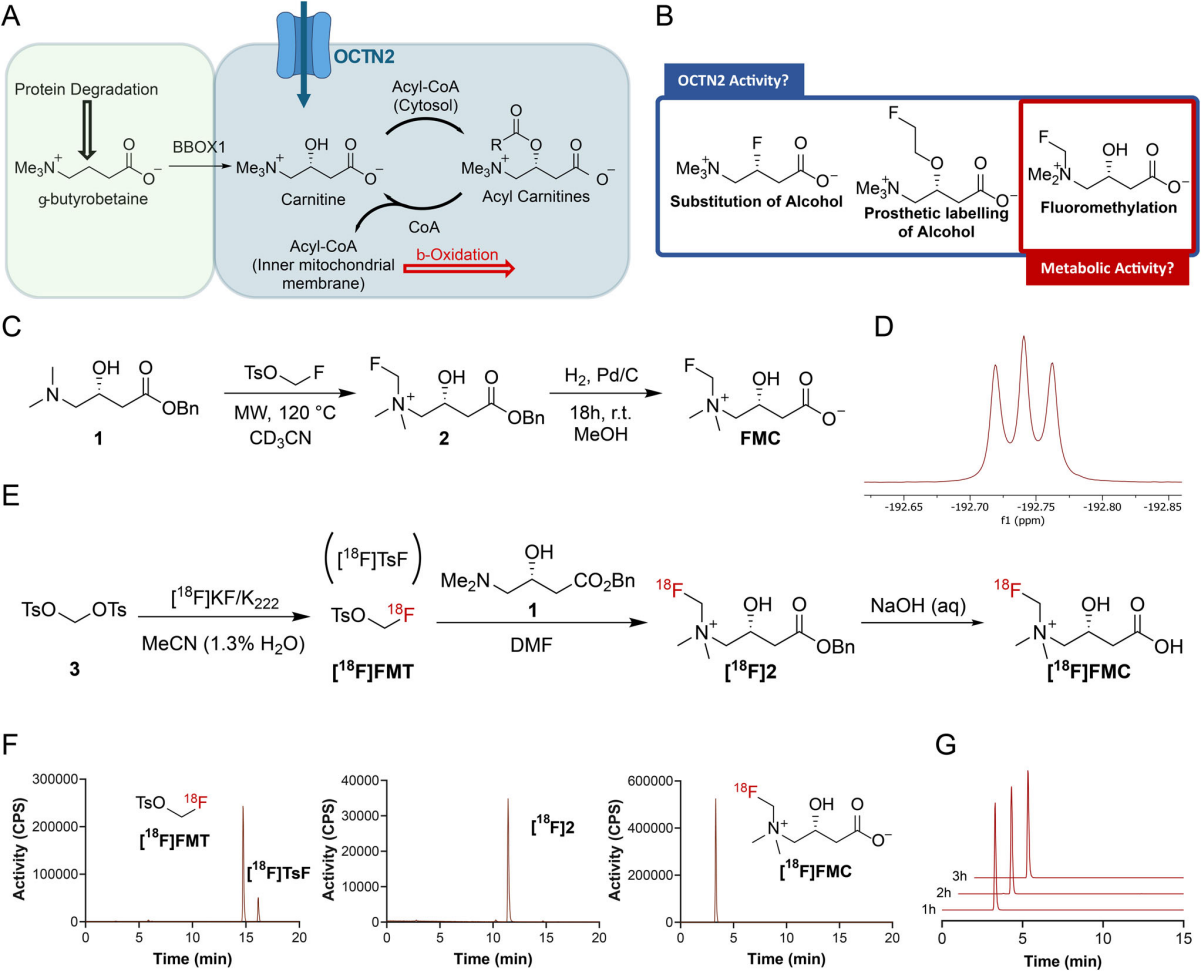
Design and synthesis of ^19^F‐ and ^18^F‐labelled carnitine analogues. A) Carnitine metabolism; *de novo* synthesis of carnitine (green) and the carnitine shuttle (blue). B) Methods for introducing fluorine to carnitine's structure and the predicted effect on its transport and metabolism. C) Synthesis of FMC. D) Proton decoupled ^19^F NMR of FMC shows a characteristic scalar coupling ^2^J_FN_ to the spin‐1 ^14^N nucleus. E) Radiosynthesis of [^18^F]FMC. F) Representative radio‐HPLC chromatograms of isolated [^18^F]FMT (left), benzyl protected intermediate, [^18^F]2 (middle) and [^18^F]FMC (right). G) Radio‐HPLC chromatograms showing formulation stability of [^18^F]FMC in saline at room temperature.

ʟ‐carnitine and its acyl derivatives are essential for a variety of biological functions and metabolic pathways,^[^ ^1^, ^2^, ^3^ ^]^ the most important of which is the transport of fatty acids into the mitochondria for β‐oxidation, producing ATP (**Figure** **1**A).^[^ ^1^, ^4^ ^]^ This process, known as ‘the carnitine shuttle’, proceeds via conjugation of fatty acids to carnitine by carnitine palmitoyltransferase (CPT1) on the outer mitochondrial membrane to generate acyl‐carnitines. Subsequent carnitine‐mediated transport of these fatty acids to the inner mitochondrial matrix and, finally, cleavage of the acyl‐carnitine ester by CPT2 provides the fatty acid for β‐oxidation and liberates free carnitine, which is recycled to continue the shuttling process.^[^ ^1^, ^4^ ^]^ Cellular carnitine homeostasis is regulated by its *de novo* synthesis from gamma‐butyrobetaine by gamma‐butyrobetaine dioxygenase 1 (BBOX1) or by transport via the sodium‐dependent cation transporter, OCTN2.^[^ ^2^, ^4^ ^]^

In addition to its vital role in facilitating β‐oxidation, carnitine regulates acyl‐CoA:CoA ratios, eliminates irregular organic acids, and acts as an antioxidant and cellular protectant.^[^ ^1^, ^5^ ^]^ Concentrations of (acyl)carnitines and the ratio between ‘free’ carnitine and its acyl derivatives are widely used to identify inborn errors of fatty acid metabolism.^[^ ^2^ ^]^ Additionally, altered carnitine transport and aberrant carnitine metabolism are associated with a variety of diseases and disorders, including heart disease,^[^ ^2^ ^]^ cancer^[^ ^6^, ^7^ ^]^ and insulin resistance.^[^ ^2^ ^]^ Carnitine has been established as an effective treatment in some cases, including for primary carnitine deficiency.^[^ ^1^, ^2^ ^]^

Despite carnitine's established importance for healthy energy metabolism, a quarter of a billion USD supplement market, and its approved application as both a pharmaceutical and a disease biomarker, there remains debate over the value and risks of carnitine supplementation.^[^ ^2^, ^8^, ^9^ ^]^ Indeed, mixed clinical results have been observed with carnitine supplementation in the context of Alzheimer's disease and dementia,^[^ ^10^, ^11^, ^12^, ^13^ ^]^ cardiovascular disease (CVD) and peripheral artery disease,^[^ ^14^, ^15^, ^16^ ^]^ diabetes and insulin resistance,^[^ ^17^, ^18^ ^]^ infertility,^[^ ^19^, ^20^ ^]^ osteoarthritis,^[^ ^21^, ^22^ ^]^ and athletic performance enhancement.^[^ ^23^, ^24^ ^]^ New tools and techniques are therefore required to better understand healthy, supplemented, and dysregulated carnitine utilisation.

Fluorinated analogues of endogenous small molecules, such as amino acids and sugars, have proven to be invaluable tools for visualising metabolic processes.^[^ ^25^, ^26^, ^27^, ^28^ ^]^ Labelling biomolecules with fluorine enables detection, identification, and quantification of the parent compound and its downstream metabolites using ^19^F nuclear magnetic resonance (NMR) and mass spectrometry (MS).^[^ ^29^, ^30^, ^31^ ^]^ Both ^19^F‐NMR and MS rely on the low abundance of fluorine in living systems to provide high signal‐to‐noise detection of the exogenously administered ^19^F‐labelled species within the complex biological milieux. The biotransformation of the labelled molecule can subsequently be identified and measured, enabling the interrogation of specific metabolic pathways.

The use of ^18^F‐labelled biomolecules for in vivo imaging with positron emission tomography (PET) has had a considerable impact on our understanding of healthy and perturbed metabolism, as well as our ability to diagnose diseases.^[^ ^25^, ^26^, ^32^, ^33^, ^34^, ^35^ ^]^ [^18^F]2‐fluoro‐2‐deoxy‐D‐glucose (FDG) PET is perhaps the best example, providing a readout of aberrant glucose utilisation and used clinically for the staging and restaging of cancer.^[^ ^36^ ^]^ Building on this prior work, we hypothesized that the generation of an appropriately designed (radio)fluorinated carnitine derivative would provide a valuable molecular probe to interrogate carnitine metabolism, as well as a diagnostic tracer for pathologies exhibiting abnormal carnitine utilisation. Toward this goal, we developed a strategy that enables the labelling of carnitine with fluorine‐19 or fluorine‐18 without perturbing its biological activity. The strategy was inspired by the synthesis of [^18^F]fluoromethylcholine ([^18^F]FCH), a radiotracer with widespread utility for cancer imaging.^[^ ^37^ ^]^

Herein, we report the design and synthesis of fluoromethylcarnitine (FMC) and its radiolabelled derivative, [^18^F]FMC, and demonstrate their ability to visualise carnitine transport and metabolism both in vitro and in vivo. Furthermore, carnitine utilisation in cancer was imaged for the first time with [^18^F]FMC PET in a xenograft model of non‐small cell lung cancer (NSCLC), revealing increased carnitine utilisation in the transformed cells compared to healthy lung tissue.

## 2 Results and Discussion

### 2.1 Probe Design and Synthesis

Fluorinated carnitine probes were designed so that the addition of the fluorine atom would not perturb carnitine transport or its downstream metabolism. Carnitine is a small zwitterionic molecule bearing both a carboxylate and trimethyl ammonium moiety, which facilitates its transport via OCTN2, and a secondary alcohol that facilitates its acylation.^[^ ^38^, ^39^ ^]^ Substitution of carnitine's secondary alcohol with fluorine or functionalisation of the alcohol with a fluorine‐labelled prosthetic was considered synthetically feasible and would maintain the probe's recognition by OCTN2. Indeed, several prodrug strategies have relied on the modification of carnitine via its alcohol.^[^ ^40^, ^41^, ^42^ ^]^ However, the secondary alcohol is essential for conjugating carnitine to fatty acids for subsequent transport into the mitochondria. Modification of the alcohol was therefore considered unfeasible if normal metabolic activity was to be maintained, whilst any alterations at the trimethyl ammonium or the carboxylate would need to preserve carnitine's zwitterionic nature. We hypothesized that the introduction of fluorine at the trimethyl ammonium would have the lowest impact on the carnitine probe's biological activity, maintaining all functionality required for both transport and metabolism (Figure 1B).

To incorporate fluorine at the trimethylammonium moiety, fluoromethylation of the dimethylamine is a promising approach. Rydzic et. al. have previously reported the synthesis of a fluorinated derivative of γ‐butyrobetaine, the *de novo* precursor to carnitine, by fluoromethylation.^[^ ^29^ ^]^ However, the diarylsulfonium fluoromethylating reagent employed requires a 2‐step synthesis and is not applicable to radiosynthesis. Radiofluoromethylation using [^18^F]fluoromethyltosylate ([^18^F]FMT) is a well‐established method for the incorporation of fluorine‐18 into biomolecules and is used clinically to produce [^18^F]FCH.^[^ ^37^, ^43^, ^44^, ^45^ ^]^ Conversely, the use of FMT as a fluoromethylating reagent for standard synthesis is underexplored.^[^ ^46^, ^47^ ^]^ Here, we sought to develop a method employing FMT that could rapidly generate both fluorinated and radiofluorinated carnitine derivatives.

Adapting a procedure reported by Brocklesby et al., we produced FMT on a gram scale.^[^ ^46^ ^]^ Initial attempts to fluoromethylate norcarnitine with FMT using similar conditions used for the radiosynthesis of [^18^F]FCH were unsuccessful (**Table** **1**, entry 1). However, fluoromethylation of its benzyl ester (**1**) proved fruitful (Figure 1C), although conversion was low (Table 1, entry 2). Promisingly, the fluoromethylated intermediate (**2**) was isolated in high purity using cartridge‐based solid‐phase extraction (SPE), a technology often used to expedite the isolation of radiopharmaceuticals. Proton decoupled ^19^F‐NMR analysis showed a triplet due to the ^2^J_FN_ scalar coupling to the spin‐1 ^14^N nucleus, characteristic of the symmetric tetrahedral geometry at the quadrupolar ^14^N nucleus generated by the formation of the fluorinated quaternary ammonium (Figure 1D).^[^ ^29^ ^]^ Encouraged by these results, we looked to increase the conversion to **2** by increasing the temperature and duration of the reaction (Table 1, entries 3 and 4). Whilst conversion was improved, this came at the cost of final product purity, with protected carnitine (**4**) detectable in increasing amounts by ^1^H‐NMR (Table 1, entries 3 and 4). The mechanism underlying the defluorination of compound **2** to form by‐product **4** remains unclear. The presence of a formal positive charge on the adjacent quaternary ammonium group makes the loss of fluorine as F^−^ unlikely. A radical mechanism, involving subsequent proton abstraction by the quaternary ammonium radical, appears more plausible; however, further studies are required to elucidate the precise decomposition pathway.

**Table 1:** Optimisation of the fluoromethylation reaction with FMT.

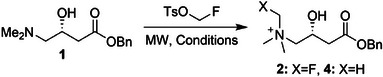						
Entry	Solvent, Conditions^a)^	FMT (eq)	^19^F‐NMR conversion [%]	Isolated yield [%]	Product ratio ^b)^	
					2 [%]	4 [%]
1^c)^	CD_3_CN, 120 °C, 0.5 h	1.5	0	–	–	–
2	CD_3_CN, 120 °C, 0.5 h	1.5	Trace	<5%	95	5
3	CD_3_CN, 120 °C, 4 h	1	39	15	84	16
4	CD_3_CN, 140 °C, 4 h	1.5	65	66	70	30
5	DMF, 120 °C, 4 h	1	–	16	92	8
6	DMA, 120 °C, 4 h	1	–	18	93	7
7	CD_3_CN, 120 °C, 4 h	4	–	25	98	2

^a)^ Reactions were performed on a 0.19–0.38 mmol scale.

^b)^ Determined by proton NMR.

^c)^ Norcarnitine (free carboxylate) used as the starting material instead of **1**. MW, microwave.

As the separation of **4** from its fluorinated derivative (**2**) is extremely challenging and not achievable using SPE purification, we hoped to reduce the protected carnitine impurity by modulating the fluromethylation conditions. Both DMA and DMF were suitable solvents for the fluoromethylation reaction, but did not sufficiently reduce the amount of **4**. However, by increasing the number of equivalents of FMT and shortening the reaction time (Table 1, entry 7), **2** could be obtained in high purity (98%) and in good yield (25%) for subsequent deprotection, to provide high‐purity FMC in 22% yield in three steps (Figure 1C). For the full optimisation of the fluoromethylation reaction, please refer to Table S1 (Supporting Information).

We next explored the most suitable method to produce [^18^F]FMT, which was required for the radiosynthesis of [^18^F]FMC (Figure 1F). Synthesis of the radiofluoromethylating reagent proceeds via S_N_2 reaction of nucleophilic [^18^F]fluoride at the methylene of ditosylmethane. However, the process needs to be carefully tuned to minimise nucleophilic attack at the sulphur of the tosylate, which generates undesirable [^18^F]tosylfluoride ([^18^F]TsF) side product.^[^ ^48^ ^]^ A range of reported methods were tested to obtain [^18^F]FMT in high radiochemical yield (RCY) with minimal [^18^F]TsF (see Table S2, Supporting Information). A method adapted from Smith et al.^[^ ^49^ ^]^ was taken forward, producing [^18^F]FMT in excellent isolated RCY (85 ± 7.1%, *n* = 6) with acceptable amounts of [^18^F]TsF impurity (31 ± 12%, *n* = 6).

[^18^F]FMT was isolated on a C18 light SPE cartridge and dried by passing a flow of N_2_ through the cartridge. Once dry, [^18^F]FMT was eluted with a solution of **1** in DMF for the key fluoromethylation reaction. The reaction conditions and the weak cation exchange (WCX) SPE purification, optimised for the ‘cold’ non‐radioactive synthesis and purification of **2**, translated well to the radiosynthesis and purification of its ^18^F‐labelled counterpart (**[^18^F]2**). Radiochemical conversion (RCC) from [^18^F]FMT to **[^18^F]2** was 91 ± 5.0% (*n* = 3) using 100 mg of precursor **1**. The RCC was only slightly reduced by halving the precursor amount to 50 mg (76 ± 9.1% RCC, *n* = 3). RCP of **[^18^F]2** was > 95% after WCX SPE purification. Finally, **[^18^F]2** was quantitatively deprotected with NaOH (0.12 N), which was neutralised with HCL (0.12 N) to provide stable, saline‐formulated [^18^F]FMC. Chromatograms showing the different steps of the radiosynthesis are shown in Figure 1F. The final formulated product was obtained in good RCY (16.2 ± 3.2%, *n* = 8; activity yield of ≈45 MBq) over three steps, with a high RCP (> 95%) and a molar activity (A_m_) of 84.2 ± 28 MBq µmol^−1^ (*n* = 4). The relatively low A_m_ likely reflects the small‐scale, manual radiosynthesis of [^18^F]FMC and is expected to improve upon adaptation to high‐scale automated radiosynthesis – a process under development in our laboratory. The product was stable in saline for over 3 h (Figure 1G).

### 2.2 Characterisation and In Vitro Evaluation of FMC and [^18^F]FMC

**Figure 2:**
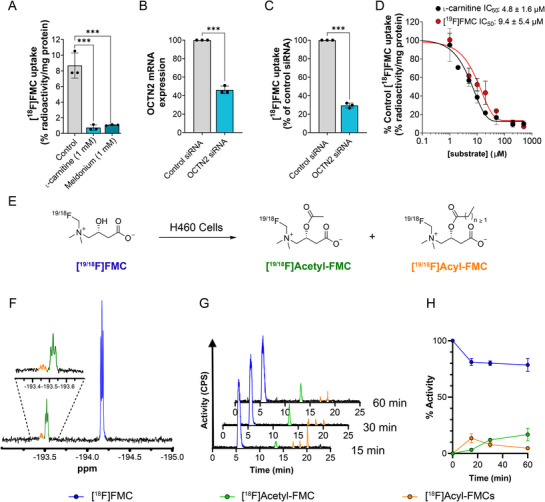
In vitro evaluation of [^18/19^F]FMC. A) Cell‐associated radioactivity in H460 cells following incubation with [^18^F]FMC in the presence and absence of ʟ‐carnitine and OCTN2 inhibitor, meldonium (*n* = 3). B) OCTN2 mRNA levels in H460 NSCLC cells following OCTN2 siRNA treatment. Differences in mRNA expression were compared to control siRNA‐treated cells (*n* = 3). C) [^18^F]FMC cell uptake after incubation with H460 cells following OCTN2 siRNA treatment. Differences in [^18^F]FMC cell uptake were compared to control siRNA‐treated cells (*n* = 3). D) [^18^F]FMC cell uptake after incubation with H460 cells in the presence of either ʟ‐carnitine or [^19^F]FMC. E) Chemical structures of the products formed following incubation of [^18^F]FMC with H460 cells. F) ^19^F‐NMR spectra of H460 cell lysates following 24 h incubation with [^19^F]FMC. G) Representative radio‐HPLC chromatograms of cell‐associated radioactivity following incubation of [^18^F]FMC with H460 cells. H) Quantification of the % radioactivity corresponding to different radioactive species following incubation of [^18^F]FMC with H460 cells over time (*n* = 4‐5). Data are presented as mean ± SD. For A, 1‐way analysis of variance (ANOVA) followed by t‐tests multiple comparison correction (Dunnett's method) was performed. For B‐C, an unpaired two‐tailed Student's *t*‐test with Welch's correction was performed. ^*^ *p* < 0.001; ^**^ *p* < 0.005; ^***^ *p* < 0.0005.

Once robust syntheses had been established for the ^19^F‐ and ^18^F‐labelled carnitines, we assessed their respective biological activities. To determine whether [^18^F]FMC was still transported into cells via OCNT2, radiotracer uptake assays were performed using the H460 human non‐small cell lung cancer (NSCLC) cell line. Incubation of [^18^F]FMC with H460 cells resulted in high cellular uptake of the radiotracer (8.7 ± 1.6% radioactivity per mg protein, *n* = 3), which, importantly, was blocked by co‐incubation of its natural substrate, ʟ‐carnitine (84.3 ± 9.8% reduction), or a specific OCTN2 inhibitor, meldonium (78.1 ± 10.2% reduction; **Figure** **2**A). To further validate the transporter specificity of [^18^F]FMC for OCTN2, siRNA knockdown of OCTN2 in H460 cells was performed, as confirmed by qPCR. Uptake of [^18^F]FMC was proportionally reduced in the H460 cells transfected with OCTN2 siRNA versus the siRNA control (Figure 2B,C). Having confirmed that [^18^F]FMC maintained specificity for OCTN2, we sought to establish if the replacement of the original proton with a fluorine atom affected substrate affinity. Affinity for OCTN2 was measured for both FMC and ʟ‐carnitine by competitive inhibition of [^18^F]FMC uptake at a range of concentrations in H460 cells (Figure 2D). The IC_50_ of FMC (9.4 ± 5.4 µm) was unaltered compared to carnitine (4.8 ± 1.6 µm, *p* = 0.849, *n* = 3), suggesting incorporation of fluorine at the quaternary ammonium of carnitine had no impact on OCTN2 transport. Additionally, these data suggest that the chiral integrity of FMC was maintained, as D‐carnitine exhibits a markedly lower affinity for OCTN2 compared with the ʟ‐enantiomer. Reported Michaelis–Menten constants (K_m_) for ʟ‐ and d‐carnitine are 4.8 ± 0.3 and 98.3 ± 38.0 µm, respectively.^[^ ^50^ ^]^

### 2.3 FMC and [^18^F]FMC as Tools to Analyse Carnitine Metabolism in Cells

To understand if FMC underwent typical carnitine metabolism, we incubated both FMC and [^18^F]FMC with H460 cells (Figure 2E) and analysed the lysates for the formation of fluorinated acyl‐carnitine derivatives using ^19^F‐NMR, LC‐MS, and radio‐HPLC. ^19^F‐NMR analysis of H460 cells after 24 h incubation with FMC showed the formation of new fluorinated species still bearing the fluorinated quaternary ammonium moiety, as characterised by the conservation of the proton decoupled ^14^N triplet (Figure 2F). LC‐MS analysis of the same lysates showed the presence of multiple fluorinated acyl‐carnitines (**Table** **2**).

**Table 2:** LC‐MS analysis of cell lysates incubated with FMC.

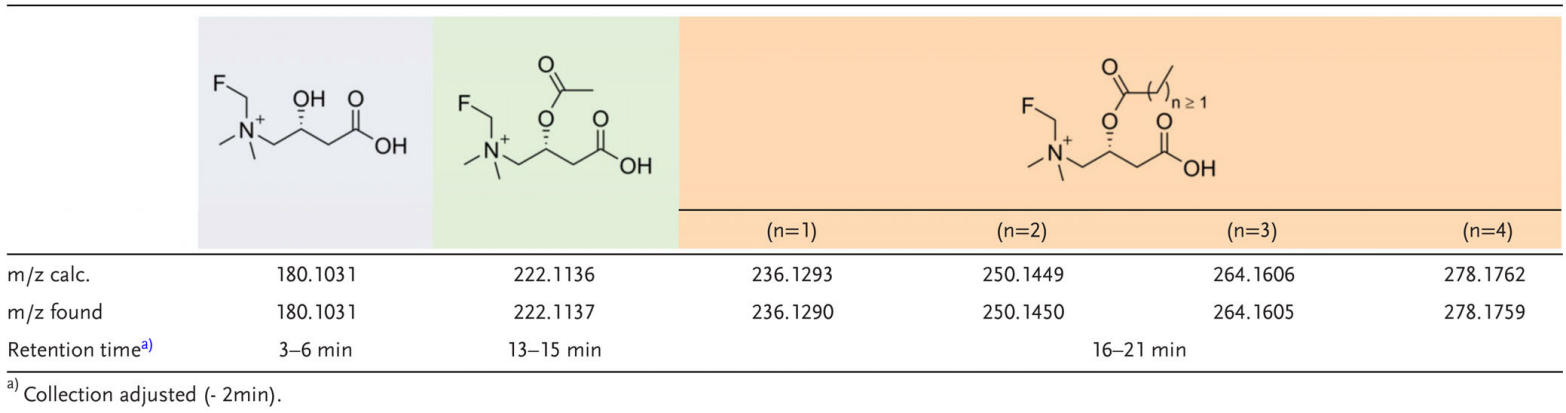

Cell extracts from H460 cells incubated with [^18^F]FMC and analysed by radio‐HPLC revealed multiple metabolites more lipophilic than [^18^F]FMC itself (Figure 2G). The retention times of the radio‐metabolites corresponded with fluorinated acetyl‐carnitine and longer‐chain fatty acid derivatives detected using LC‐MS (Table 2). Time course evaluation of [^18^F]FMC metabolism (Figure 2H) revealed rapid conversion to a mixture of corresponding acyl‐carnitines within 15 min, and a time‐dependent increase in acetyl‐carnitine ([^18^F]acetyl‐FMC). Under physiological conditions, acetyl‐carnitine makes up most of the acyl‐carnitine pool. It is therefore probable that the [^18^F]FMC metabolite distribution is moving toward steady‐state equilibrium of (acyl)carnitines present within the cell. Taken together, the results indicate both FMC and [^18^F]FMC undergo normal carnitine transport across both the plasma and mitochondrial membranes, where they undergo metabolic conversion. Consequently, FMC and [^18^F]FMC can be employed as tools to measure the dynamics of these biological processes.

### 2.4 Visualising Carnitine Utilisation In Vivo with [^18^F]FMC

**Figure 3:**
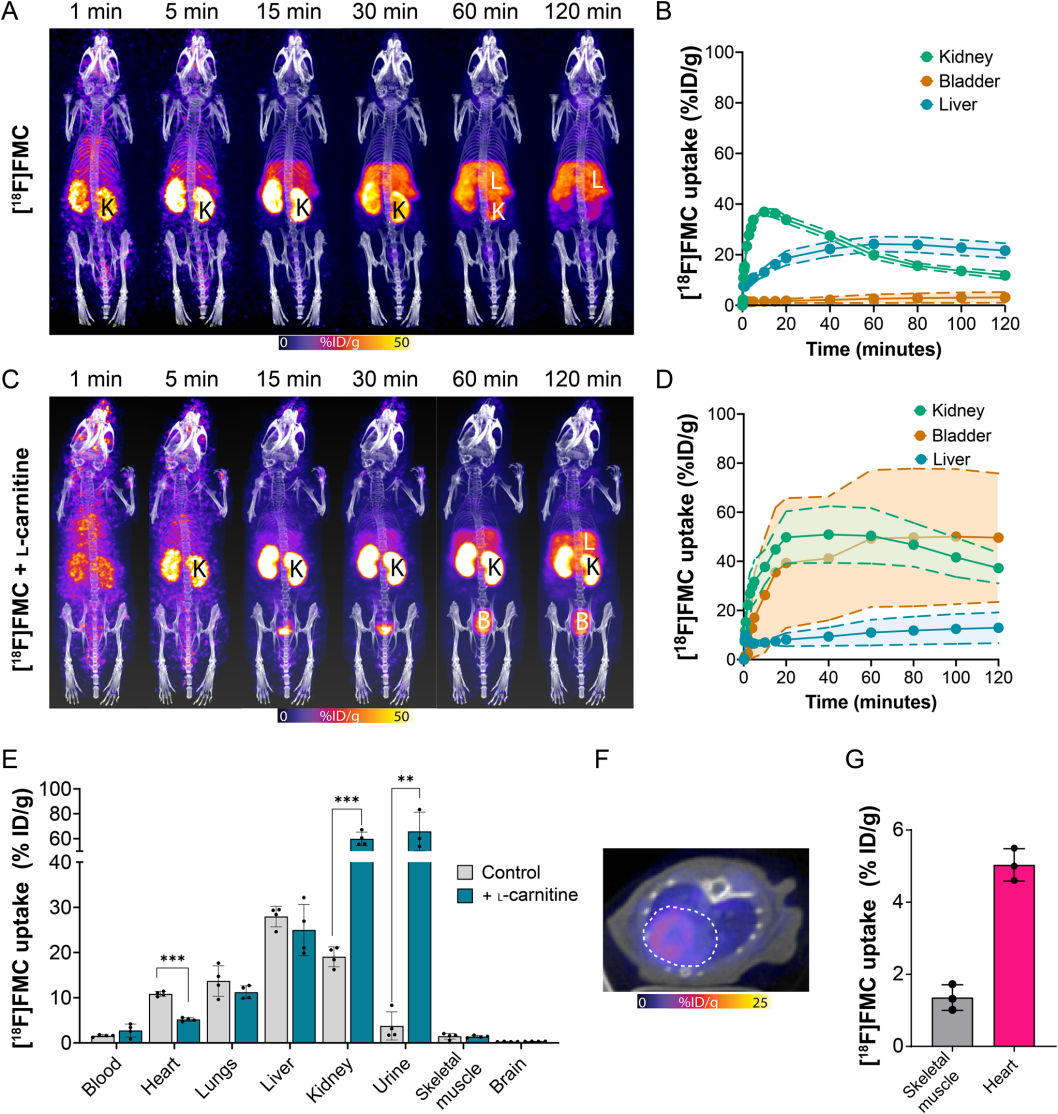
Imaging carnitine utilisation in vivo with [^18^F]FMC PET. A) Representative 1–120 min maximum intensity projection PET/CT images of healthy Balb/C mice following tail vein injection of [^18^F]FMC (*n* = 4). B) Image‐derived time activity curves (TAC) for the kidney, liver, and bladder from a dynamic 120 min scan following i.v. injection of [^18^F]FMC (*n* = 4). C) Representative 1–120 min maximum intensity projection PET/CT images of healthy Balb/C mice following tail vein injection of [^18^F]FMC plus 400 µmol ʟ‐carnitine (*n* = 4; K, kidney; B, bladder; L, liver). D) Image‐derived TAC for kidney, liver, and bladder from a dynamic 120 min scan and following i.v. co‐injection of [^18^F]FMC and 400 µmol ʟ‐carnitine. Uptake is expressed as % injected dose (ID)/g. Data are mean ± SD (*n* = 4). E) Ex vivo biodistribution of [^18^F]FMC in selected tissues at 2 h post‐injection (*n* = 4). For analysis, multiple unpaired two‐tailed Student's *t*‐test with Welch's correction was performed. F) Axial slice showing [^18^F]FMC uptake in the myocardium. G) Image‐derived quantification of [^18^F]FMC uptake in the heart and skeletal muscle (*n* = 3). Data are presented as mean ± SD. ^*^ *p* < 0.001; ^**^ *p* < 0.005; ^***^ *p* < 0.0005.

Given that [^18^F]FMC mapped carnitine metabolism in cells, we next assessed its ability to non‐invasively probe carnitine utilisation in vivo. [^18^F]FMC was injected into healthy nude mice via the tail vein, and dynamic PET scans were acquired for 2 h, which was followed by biodistribution measurements ex vivo from excised tissue. [^18^F]FMC was rapidly cleared from the blood and reabsorbed by the kidneys before accumulating in the liver (**Figure** **3**A,B). The observed reabsorption reflects the well‐established mechanisms in place to maintain homeostasis of the carnitine pool.^[^ ^4^, ^51^ ^]^ In humans, > 97% of carnitine is reabsorbed by the kidneys back into the blood when kidney function and carnitine blood levels are both normal.^[^ ^52^ ^]^

To establish if [^18^F]FMC could be used to visualise disrupted kidney reabsorption, we co‐injected carnitine (4.7 mg kg^−1^) with [^18^F]FMC to generate a carnitine blood concentration of ≈400 µm – a concentration known to saturate carnitine reabsorption in the healthy kidney (≈10 times the kidney reabsorption threshold).^[^ ^52^ ^]^ Here, [^18^F]FMC was rapidly excreted to the bladder via the kidneys, which retained radioactivity up to 60 min before decreasing due to a combination of urinary excretion and reabsorption back into the blood (Figure 3C–E). The results align with clinical observations for indications characterised by reduced renal function and/or a deficiency of functional OCTN2 transporter, including primary carnitine deficiency.^[^ ^1^ ^]^ In addition to uptake in the kidney and liver, [^18^F]FMC also accumulated in the heart, which was significantly reduced by co‐injection of carnitine (Figure 3E). [^18^F]FMC accumulation in the heart (without carnitine co‐injection) showed clear delineation of the left ventricular myocardium (Figure 3F). Accumulation of [^18^F]FMC was well above background, with a heart‐to‐skeletal muscle ratio of 3.7 (Figure 3G).

It is important to note that [^18^F]FMC PET reveals the dynamic changes in carnitine uptake, retention, and redistribution rather than carnitine abundance or concentration. For example, skeletal muscle typically has the highest concentration of carnitine. Yet, minimal [^18^F]FMC uptake was observed in this tissue. While muscle carnitine levels are high, the kinetics of carnitine uptake into muscle tissue are slow.^[^ ^53^ ^]^ This is reflected in the distribution of supplemented carnitine, which has little impact on muscle carnitine concentrations,^[^ ^54^ ^]^ as well as in the [^18^F]FMC PET images.

### 2.5 Imaging Aberrant Carnitine Utilisation and Metabolism in NSCLC

Metabolic reprogramming has been established as an important hallmark of cancer.^[^ ^55^, ^56^ ^]^ Therapeutic strategies targeting metabolic vulnerabilities aim to leverage these alterations in transformed cells by inhibiting pathways key to cancer progression using targeted drugs or dietary alterations.^[^ ^57^, ^58^ ^]^ Diagnostic tools informing on metabolic phenotypes can enable patient stratification and aid in the development of new targeted therapies. Carnitine metabolism is a potential target for such treatments, with dysregulated carnitine homeostasis reported in several cancers.^[^ ^6^, ^7^, ^59^, ^60^, ^61^, ^62^ ^]^

**Figure 4:**
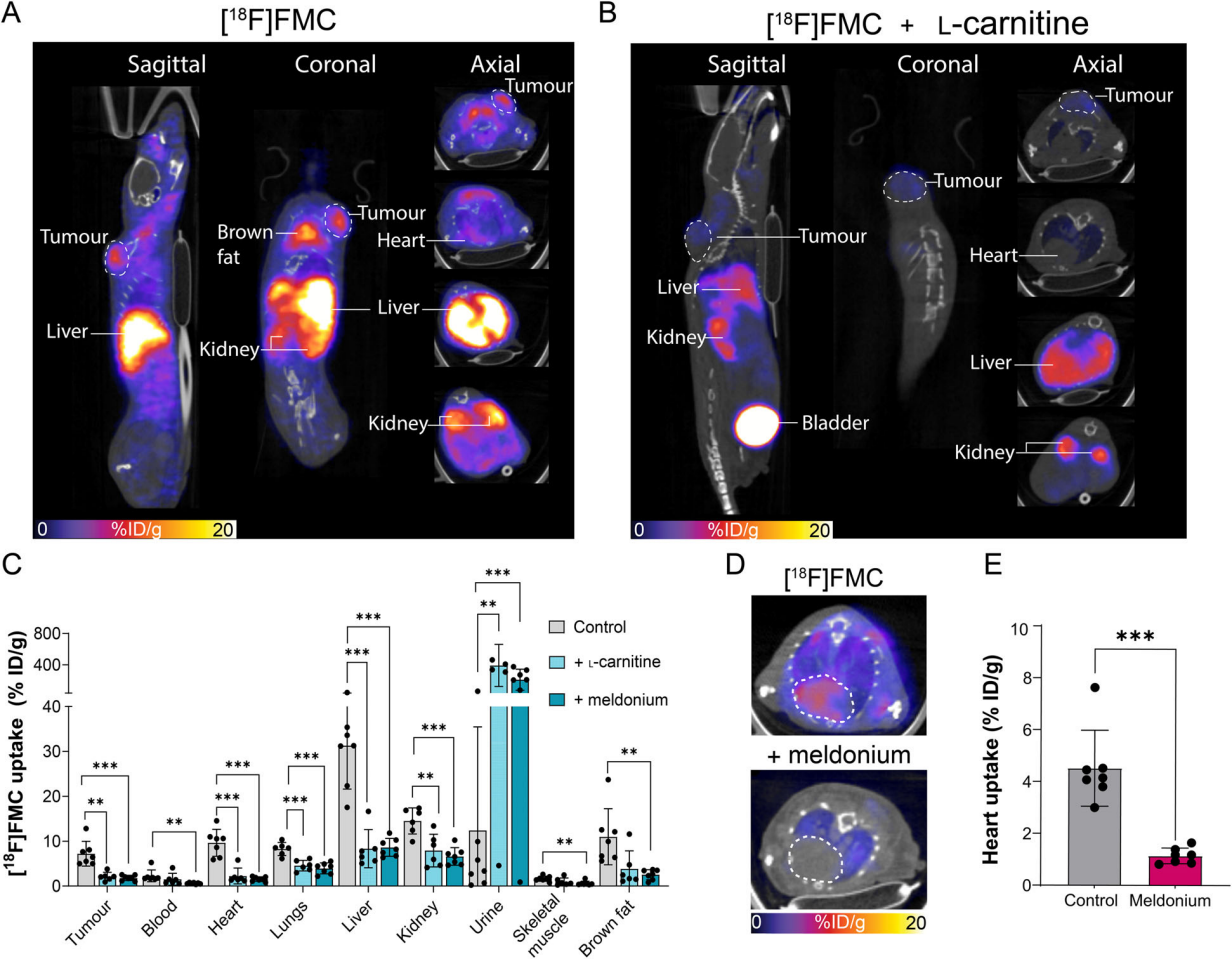
Imaging aberrant carnitine utilisation in NSCLC with [^18^F]FMC PET. A and B) Representative 120 min sagittal, coronal, and axial PET/CT images of [^18^F]FMC in a human NSCLC (H460) subcutaneous xenograft tumour model (A), with co‐injection of carnitine (50 mg kg^−1^; B). C) Ex vivo biodistribution of [^18^F]FMC in selected tissues at 2 h post‐injection in the presence and absence of carnitine and meldonium (*n* = 6‐7). Analysis was performed by one‐way analysis of variance (ANOVA) followed by *t*‐tests, multiple comparison correction (Dunnett's method). D) Representative axial PET/CT images of heart [^18^F]FMC uptake at 120 min with and without co‐injection of meldonium. An unpaired two‐tailed Student's *t*‐test with Welch's correction was performed (*n* = 7). E) Image‐derived quantification of [^18^F]FMC heart uptake at 120 min in the presence and absence of meldonium. Data are presented as mean ± SD. ^*^ *p* < 0.001; ^**^ *p* < 0.005; ^***^ *p* < 0.0005.

Given that [^18^F]FMC mirrored carnitine utilisation in healthy mice, we next explored the use of [^18^F]FMC PET to visualise aberrant carnitine utilisation in cancer. In this setting, carnitine utilisation has previously been studied using ex vivo techniques, such as MS imaging.^[^ ^63^, ^64^, ^65^, ^66^ ^]^ However, carnitine utilisation in tumours has yet to be visualised in living subjects. Here, [^18^F]FMC PET imaging was performed in H460 NSCLC tumour‐bearing mice. Healthy tissue distribution of [^18^F]FMC closely matched that observed in healthy mice, with rapid extraction by the kidneys and redistribution to the liver (**Figure** **4**). Tumour retention of [^18^F]FMC increased over the imaging time course (Figure S1, Supporting Information), with uptake in the tumour reaching 7.2 ± 2.8% ID g^−1^ at 2 h (*n *= 7). [^18^F]FMC tumour uptake was considerably higher than blood and muscle, allowing clear delineation of the H460 tumour in the PET images (Figure 4A), yielding tumour‐to‐blood and tumour‐to‐muscle ratios of 3.2 and 4.9, respectively. Tumour [^18^F]FMC uptake was blocked by co‐injection with either ʟ‐carnitine (Figure 4B) or the selective OCTN2 inhibitor, meldonium (Figure S2, Supporting Information), suggesting [^18^F]FMC tumour uptake was mediated by OCTN2. [^18^F]FMC tissue distribution was corroborated by ex vivo biodistribution analysis (Figure 4C). The dynamic imaging of carnitine utilisation in tumours provides opportunities to improve our understanding of cancer metabolism in vivo. Visualisation of tumour metabolic plasticity during nutrient restriction or therapeutic pressure using [^18^F]FMC PET is an exciting possibility.

With PET, we can assess metabolism across the entire animal, which affords additional benefits. Carnitine or meldonium co‐injection not only reduced [^18^F]FMC tumour accumulation, but also suppressed uptake in the heart, liver, kidney, and brown fat (Figure 4C–E). Meldonium, or mildronate, is used for the treatment of stable angina and has been evaluated clinically for the treatment of myocardial ischaemia.^[^ ^67^, ^68^ ^]^ The results from our study suggest that [^18^F]FMC could be used to visualise meldonium inhibition of OCTN2 transport in cardiac tissue, potentially providing a readout of treatment efficacy or aiding dose optimisation.

### 2.6 Assessing the Metabolic Fate of [^18^F]FMC

**Figure 5:**
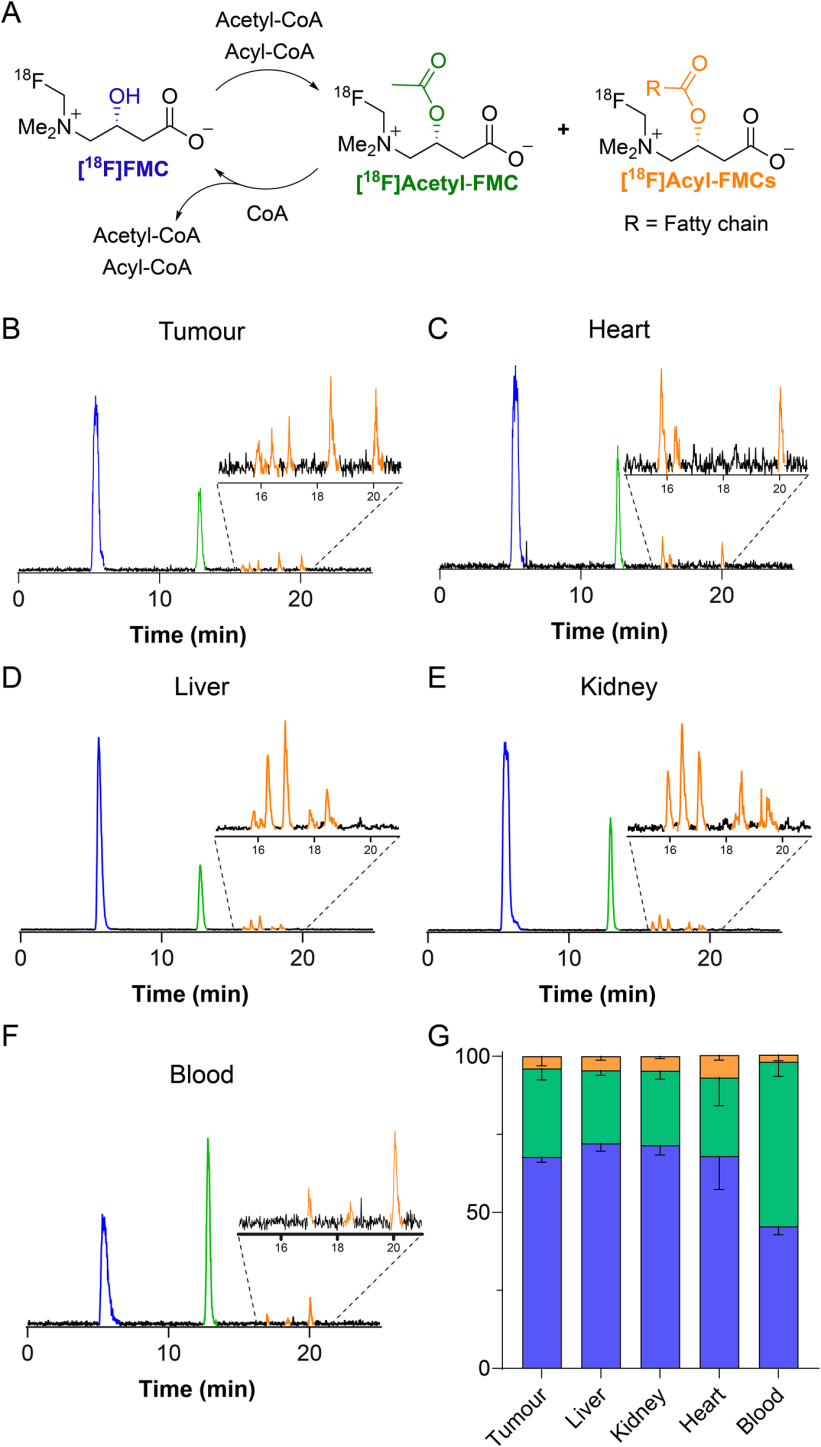
Visualising carnitine metabolism in healthy and malignant tissue with [^18^F]FMC. A) Proposed mechanism for in vivo metabolism of [^18^F]FMC. B‐F) Representative radio‐HPLC chromatograms showing the radio‐metabolite distribution of [^18^F]FMC in different tissues at 120 min post‐injection. G) Quantification of the % radioactivity corresponding to different radioactive species in different tissues at 120 min post‐injection (*n* = 4).

Having established [^18^F]FMC as a novel carnitine in vivo tracer, we next assessed the downstream metabolism of [^18^F]FMC in different tissues. Ex vivo metabolite analysis was performed on the H460 tumours, blood, and healthy tissues that exhibited high [^18^F]FMC accumulation (heart, liver, and kidney). Tissues were harvested, homogenised, and the associated radioactive components extracted for analysis using radio‐HPLC. Similarly to cell extracts, [^18^F]FMC was the dominant species in tissue homogenates. Importantly, no defluorination of the radiotracer was observed (separation of [^18^F]FMC and [^18^F]fluoride was confirmed by co‐injection; see Figure S3, Supporting Information). Lipophilic [^18^F]FMC metabolites were present in all analysed tissues, suggesting that [^18^F]FMC undergoes essential carnitine metabolism in vivo to generate both acetyl‐carnitine ([^18^F]acetyl‐FMC) and longer chain fatty acid acyl‐carnitines ([^18^F]acyl‐FMC; **Figure** **5**A). In line with the in vitro metabolite analysis, radio‐metabolites were observed at retention times corresponding to the acetyl‐ and acyl‐conjugated [^19^F]FMC derivatives (Table 2).

The percentage of [^18^F]acetyl‐FMC metabolite varied between tissues, with higher amounts observed in the blood (52.7 ± 4.7%) compared to the other tissues (kidney, 23.9 ± 2.7%; liver, 23.4 ± 1.5; tumour, 28.3 ± 3.7% and heart, 25.2 ± 9.0; Figure 5B–G; Table S3, Supporting Information). These results align with human (acyl)carnitine distributions, where acetyl‐carnitine is the major component of the acyl‐carnitine pool in both tissue and plasma.^[^ ^2^, ^69^, ^70^ ^]^ The percentage of radioactivity corresponding to [^18^F]acyl‐FMCs also varied substantially between different tissues. For example, the amount of [^18^F]acyl‐FMCs measured in the heart (7.2 ± 1.6%) was much higher than in the other tissues and over three‐fold higher than in the blood (2.1 ± 1.9%). Elevated [^18^F]FMC conjugation to fatty acids in the heart likely reflects β‐oxidation being the primary energy source in cardiac tissue.^[^ ^71^ ^]^ Interestingly, we observed distinct peak distributions in the [^18^F]acyl‐FMC component of the chromatogram for the different tissues (Figure 5B–F). Further work is required to establish whether this ‘fingerprint’ region corresponds to the distribution of fatty acids available for β‐oxidation in the different tissues.

## 3 Conclusion

We have designed and synthesised (radio)fluorinated carnitine derivatives to interrogate carnitine utilisation in biological systems and living subjects. Both ^19^F‐ and ^18^F‐labelled carnitine derivatives retained the essential biological activities of carnitine, concerning both transport and metabolism. The developed syntheses enabled quick access to the (radio)fluorinated compounds, with simple SPE purification to obtain the probes with excellent purity. [^18^F]FMC PET was able to visualise both healthy and aberrant carnitine utilisation in tissues, revealing high tumour carnitine avidity in a xenograft model of NSCLC. Furthermore, ex vivo analysis revealed differential downstream fatty acid metabolism of [^18^F]FMC across tissues. Consequently, FMC and [^18^F]FMC are poised to improve our understanding of healthy, supplemented, and aberrant carnitine metabolism. With this in mind, we are developing an automated synthesis to enable high‐activity clinical production of [^18^F]FMC.

## 4 Experimental Section

The complete Materials and Methods can be found in the Supporting Information.

### OCTN2 Silencing using RNAi

OCTN2 protein expression was modulated in culture through small inhibitory RNA (ON‐TARGETplus siRNA, Horizon discovery). H460 NSCLC cells were seeded into 6‐well plates 24 h prior to transfection with OCTN2 or control siRNA (L‐007456‐00‐0005/D‐001920‐01‐05, Horizon discovery). 25 nm of siRNA in 200 µL per well of Optimem (ThermoFisher) was prepared in tube A, whilst 200 µL per well of transfection reagent (T‐2001‐03, Horizon discovery) was prepared in tube B. Tube A and B were mixed and incubated for 5 min. The siRNA and transfection mixture was added to cells and topped up with 1600 µL of antibiotic‐free complete medium (RPMI: Thermofisher) for a total volume of 2000 µL per well and incubated for 8 h. The transfection mixture was then replaced with normal antibiotic‐free complete medium (RPMI: Thermofisher) for 72 h.

### Uptake and Blocking

0.185 MBq mL^−1^ solutions of radiotracer in fresh Hanks' Balanced Salt Solution (HBSS) prewarmed to 37 °C were prepared. Media was replaced with the radioactivity‐containing media. Plates were then incubated for 60 min at 37 °C and 5% CO_2_. For competition studies, inhibitors (meldonium) and transporter substrates (γ‐butytrobetaine, L‐carnitine, palmitoylcarnitine) were co‐incubated with [^18^F]FMC at a range of concentrations (1000 –0.1 µm) in 6 well plates for 60 min at 37 °C and 5% CO_2_. Uptake was expressed as % vehicle treated control. Following the appropriate incubation time, plates were placed on ice and washed three times with ice‐cold PBS to remove exogenous radioactivity. RIPA buffer (500 µL in 6 well plates; Fisher Scientific Ltd) was added to each well to lyse the cells, and cells were scraped for efficient cell lysis. Decay‐corrected radioactivity was determined on a gamma counter (300 µL of lysate; 2480 WIZARD2 automated gamma counter, PerkinElmer), and the remaining cell lysate was used to determine protein concentration following radioactive decay (Pierce BCA assay). To quantify radiotracer uptake, three 10 µL standard solutions of the radioactivity‐containing media were counted on the gamma counter, accounting for 1% of the added dose. Counts acquired from the gamma counter were adjusted to account for the whole 500 µL of cell lysate. Data were expressed as a percent of total radioactivity added to cells per mg of protein.

### ^19^F NMR of Cell Lysates

^19^F‐FMC at a concentration of 50 µm in RPMI was incubated with H460 cells for 24 h before harvesting. 1 mL of culture media was removed for later analysis and stored at −20 °C. Fresh samples of medium were also collected. Next, 3 mL of ice‐cold deuterated methanol (Merck) was added to each flask and kept on ice for 5–10 min. Cells were harvested by scraping the surface, and the cell/methanol suspensions were centrifuged for 5 min at 12 000 × *g* at 4 °C. The supernatant was removed and concentrated to a final volume of 540 µL in deuterated methanol. 60 µL of D_2_O buffer was added to each sample before NMR spectral acquisition. A standard of ^19^F‐FMC at a concentration of 50 µm was prepared in deuterated methanol. NMR work was performed at the UCL School of pharmacy by Dr Nikita Harvey. ^19^F nuclear magnetic resonance spectra were acquired using a vertical‐bore, ultra‐shielded Bruker (Karlsruhe, Germany) 14.1 T (600 MHz) spectrometer equipped with a QCI‐F cryoprobe, at 298 K using the Bruker zgig pulse program for ^1^H decoupling. Acquisition parameters were: 1024 scans; 4 dummy scans; 20.1 ppm spectral width; acquisition time 0.36 s; and pre‐scan delay 0.5 s. TopSpin (version 4.0.5) software was used for data acquisition and for metabolite quantification. Free induction decays (FIDs) were multiplied by a line broadening factor of 0.5 Hz and Fourier transformed, phase, and automatic baseline corrected.

### Mass Spectrometry

The samples prepared for ^19^F NMR analysis (see above) were fractionated by HPLC (25 × 1 min fractions). Eclipse XDB‐C18, 9.4 × 250 mm, 5 mm HPLC column at room temperature; solvent A: H_2_O (0.1% TFA), solvent B: MeOH (0.1% TFA); flow rate: 3.5 mL min^−1^; UV detectors: 254 and 190 nm; gradient: 0% B, 0–3 min; 0–5% B, 3–11 min; 5–95% B, 11–20 min; 95–0% B, 20–25 min, 5.0 mL injection loop. The samples were analysed using a Waters G2‐XS QTof with a Waters Acquity I class UPLC. Waters ACQUITY UPLC BEH C18, 1.7 µm, 2.1 mm x 50 mm HPLC column 60 °C; solvent A: H_2_O (0.1% FA), solvent B: MeCN (0.1% FA); flow rate: 0.4 mL min^−1^; +ve ionisation mode; gradient: 2% B, 0–1 min; 2–75% B, 1–4.5 min; 75–95% B, 4.5–4.6 min; 95% B, 4.6–5.6 min; 95–2% B, 5.6–5.7 min; 2% B, 5.7–6.5 min, Capillary: 3.0 kV, Sampling Cone: 40.0, Source Temperature: 120 °C, Desolvation Temperature: 250 °C, Desolvation Gas Flow: 600.0 L Hr^−1^.

### Cell Metabolism with Radio‐HPLC

H460 cells were seeded in a 10 cm^3^ cell culture dish 24 h prior to experiment. 0.5 MBq Ml^−1^ solutions of radiotracer in 5 mL of fresh Hanks' Balanced Salt Solution (HBSS) prewarmed to 37 °C were prepared. Media was replaced with the radioactivity‐containing media. Plates were then incubated for 15, 30, and 60 min at 37 °C and 5% CO_2_ before being placed on ice and the exogenous radioactivity removed. The cells were washed three times with ice‐cold PBS (3 × 5 mL) and then lysed with MeOH (5 mL) and scraped. The supernatant was transferred to a glass vial through a Millex 0.2 µm filter (Millipore, Billerica, MA, USA). The samples were evaporated at room temperature using an Asynt smart evaporator, diluted with 2 mL mobile phase (H_2_O, 0.1% TFA), passed through another Millex 0.2 µm filter (Millipore, Billerica, MA, USA), and monitored by reverse phase HPLC. Eclipse XDB‐C18, 9.4 × 250 mm, 5 mm HPLC column at room temperature; solvent A: H_2_O (0.1% TFA), solvent B: MeOH (0.1% TFA); flow rate: 3.5 mL min^−1^; UV detectors: 254 and 190 nm; gradient: 0% B, 0–3 min; 0–5% B, 3–11 min; 5–95% B, 11–20 min; 95‐0% B, 20–25 min, 2.0 mL injection loop).

### Tumour Models

For imaging, 3 × 10^6^ H460 cancer cells in 100 µL PBS were injected subcutaneously into female Balb/C nu/nu mice aged 6–9 weeks (Charles River Laboratories). Tumour growth was monitored using an electronic calliper and the volume calculated using the following equation: volume = ((π/6) × h × w × l), where h, w, and l represent, height, width, and length, respectively. Tumour size was monitored daily, with studies taking place when tumour volume reached ≈100 mm^3^. All animal experiments were performed in accordance with the United Kingdom Home Office Animal (scientific procedures) Act 1986. PPL licence used was number I27111203.

### In Vivo Imaging

For all imaging studies, mice were maintained under anaesthesia with isoflurane (1.5–2% in O2) at 37 °C during tail vein cannulation and imaging. For healthy imaging, dynamic PET scans were acquired on a Mediso NanoScan PET/CT system (1–5 coincidence mode; 3D reconstruction; CT attenuation‐corrected; scatter corrected) using the four‐bed mouse hotel.^[^ ^72^ ^]^ Images were acquired for 120 min following a bolus intravenous injection of [^18^F]FMC (≈1.5–3 MBq in 100 µL) through a tail vein cannula. For the LC co‐injection study, 400 µm of LC (Sigma) was simultaneously injected in 50 uL through a tail vein cannula. To determine radiotracer specificity in H460 tumour models, LC (50 mg kg^−1^; *n* = 4/group) and meldonium (250 mg kg^−1^; *n* = 4/group) were co‐injected i.v. with [^18^F]FMC. CT images were obtained for anatomical reference and attenuation correction (180 projections; semicircular acquisition; 50 kVp; 300 ms exposure time). The acquired data were reconstructed into 15 bins of 4 × 15 s, 4 × 60 s, and 3 × 300 s, 4 x 20 min (Tera‐Tomo 3D reconstructed algorithm; 4 iterations; 6 subjects; 400–600 keV; 0.3 mm^3^ voxel size). VivoQuant software (v 2.5, Invicro Ltd.) was used to analyse the reconstructed images. Regions of interest (ROIs) were drawn manually using CT images and 120‐min dynamic PET images. Time verses radioactivity curves (TACs) were generated using the percentage injected dose per mL (%ID/g).

### Biodistribution

Approximately ≈1.5 MBq of radiotracer was injected via the tail vein of conscious healthy mice (*n* **=** 4 per group). After 120 min, animals were sacrificed, and organs and tissues of interest were collected and weighed. The amount of radioactivity in each tissue was determined with the gamma counter to calculate uptake as % injected dose per g of wet weight tissue (ID/g).

### In Vivo Metabolite Analysis

In vivo metabolism of [^18^F]FMC was performed by radio‐HPLC analysis. Metabolites and parent tracer were quantified based on the area under the curve (region of interest) for [^18^F]FMC and its corresponding metabolites ([^18^F]Acetyl‐FMC and [^18^F]Acyl‐FMCs) and expressed as a percentage (mean ± SD). Tumour, heart, blood, liver, and kidney samples were analysed at 120 min post‐injection of the tracer.

H460 cells (3 × 10^6^) in PBS were injected into the flank of female Balb/c nu/nu mice. When the tumors reached ≈100 mm^3^ mice were anesthetized with isoflurane (1.5–2% in oxygen) and injected with ≈5 MBq of radiotracer through a tail vein cannular. Mice were maintained at 37 °C under anesthesia throughout radiotracer uptake. At 2 h p.i., the mice were sacrificed by exsanguination via cardiac puncture under terminal anaesthesia. Tumour, heart, blood, liver, and kidney samples were harvested and placed on ice prior to processing. The blood samples were centrifuged (2000 × g for 5 min, 4 °C), the plasma was removed and transferred to a 1.5 mL Eppendorf. Ice‐cold MeOH (1 mL) was added to the plasma, and the sample was briefly mixed on a Vortex. On ice, ice‐cold MeOH (1 mL) was added to the heart, liver, tumour, and kidney samples prior to homogenization using a PRECELLYS 24 tissue homogenizer. All samples were then centrifuged (12 000 × g for 5 min, 4 °C) and the supernatant transferred to a glass vial through a Millex 0.2 µm filter (Millipore, Billerica, MA, USA). The samples were evaporated at room temperature using an Asynt smart evaporator, diluted with 2 mL mobile phase (H_2_O, 0.1% TFA), passed through another Millex 0.2 µm filter (Millipore, Billerica, MA, USA), and monitored by reverse phase HPLC. Eclipse XDB‐C18, 9.4 × 250 mm, 5 mm HPLC column at room temperature; solvent A: H_2_O (0.1% TFA), solvent B: MeOH (0.1% TFA); flow rate: 3.5 mL min^−1^; UV detectors: 254 and 190 nm; gradient: 0% B, 0–3 min; 0–5% B, 3–11 min; 5–95% B, 11–20 min; 95–0% B, 20–25 min, 2.0 mL injection loop, retention time [^18^F]FMC = 5.55 min).

### Synthesis–General Information

Commercially available starting materials were purchased from Sigma–Aldrich, Alfa Aesar, and Apollo Scientific and were used without further purification. Norcarnitine was purchased from BioServUK. Solvents were obtained from Sigma–Aldrich; unless stated otherwise, reagent grade solvents were used for reactions and column chromatography. Unless otherwise specified, ‘water’ refers to sterile ultrapure water (18.2 mΩ‐cm). Reaction progress was monitored by thin‐layer chromatography (TLC) on aluminium sheets coated with silica gel 60 F254 (Merck Millipore), and detection was carried out using UV light (325 and 254 nm) and/or chemical solutions. Crude reaction mixtures were purified by automated flash column chromatography (Biotage Isolera One). Microwave reactions were performed using a CEM Discovery SP microwave synthesiser. ^1^H, ^13^C, and ^19^F Nuclear Magnetic Resonance (NMR) spectra were recorded on a Bruker Avance 400 equipped with a BBFO probe at room temperature. ^13^C NMR experiments were proton decoupled. ^1^H and ^13^C NMR spectra were reported relative to the internal reference of the relative deuterated solvent. Chemical shifts (d) were reported in ppm, and coupling constants (J) were given in Hertz (Hz). Multiplicity was described with (s): singlet, (d): doublet, (t): triplet, and (q): quadruplet. High resolution mass spectrometry data were recorded on a Waters G2‐XS QTof with a Waters Acquity I class UPLC. Waters ACQUITY UPLC BEH C18, 1.7 µm, 2.1 mm x 50 mm HPLC column 60 °C; solvent A: H_2_O (0.1% FA), solvent B: MeCN (0.1% FA); flow rate: 0.4 mL min^−1^; +ve ionisation mode; gradient: 2% B, 0–1 min; 2–75% B, 1–4.5 min; 75–95% B, 4.5–4.6 min; 95% B, 4.6–5.6 min; 95–2% B, 5.6–5.7 min; 2% B, 5.7–6.5 min, Capillary: 3.0 kV, Sampling Cone: 40.0, Source Temperature: 120 °C, Desolvation Temperature: 250 °C, Desolvation Gas Flow: 600.0 L Hr^−1^.

### Synthesis–Benzyl (R)‐4‐(dimethylamino)‐3‐hydroxybutanoate (1)

(*R*)‐4‐(dimethylamino)‐3‐hydroxybutanoic acid (norcarnitine; 2.0 g, 1.0 Eq, 13.6 mmol) was dissolved in benzyl alcohol (50.0 mL) to give a colourless suspension. Thionyl chloride (1.94 g, 1.19 mL, 1.2 Eq, 16.3 mmol) was added dropwise. The reaction was stirred at 70 °C for 2 h under N_2_. The solution was then allowed to cool to room temperature, and the volatiles were removed under reduced pressure. The residue was treated with 50 mL of HCl (0.5 N) and washed with ether (3 × 30 mL). Saturated sodium bicarbonate solution was added to the aqueous layer to adjust the pH to 8.0–8.5. The aqueous solution was then continuously extracted with dichloromethane (100 mL) for 2 h. The organic layer was dried with anhydrous Na_2_SO_4_ and concentrated to give benzyl (*R*)‐4‐(dimethylamino)‐3‐hydroxybutanoate (**1**) (2.59 g, 10.91 mmol, 80.3 %) as a colourless oil. ^1^H NMR (400 MHz, CDCl_3_) δ 7.43–7.23 (m, 5H), 5.17 (s, 2H), 4.21–4.06 (m, 1H), 2.59–2.44 (m, 2H), 2.42–2.20 (m, 2H), 2.28 (s, 6H). ^13^C NMR (101 mHz, CDCl_3_) δ 171.55, 135.85, 128.57, 128.25, 128.24, 66.40, 64.64, 64.39, 45.59, 39.84.

### Synthesis–(R)‐4‐(benzyloxy)‐N‐(fluoromethyl)‐2‐hydroxy‐N,N‐dimethyl‐4‐oxobutan‐1‐aminium formate (2)

A mixture of benzyl (*R*)‐4‐(dimethylamino)‐3‐hydroxybutanoate (**1**) (45 mg, 1.0 Eq, 190 µmol) and FMT (194 mg, 5.0 Eq, 950 µmol) in CD_3_CN (2.0 mL) was added to a microwave reaction tube (2.0–5.0 mL). The mixture was then heated by microwave irradiation at 120 °C for 1 h. The reaction mixture was allowed to cool to room temperature before being diluted with water (20 mL) and CH_2_Cl_2_ (20 mL). The two layers were mixed thoroughly and partitioned using a separating funnel. The aqueous layer was divided into two and each portion was passed through a WCX cartridge (preconditioned with 5% aqueous NH_4_OH). The WCX cartridges were then washed consecutively with aqueous NH_4_OH (5%, 10 mL), EtOH (10mL), and aqueous formic acid (1%, 10 mL). The product **2** was eluted into a round bottomed flask with a solution of formic acid in ethanol (2%, 10 mL). The volatiles were removed to give **2** as it's formic acid salt (15 mg, 190 µmol, 25%) as a colourless oil. ^1^H NMR (400 mHz, D_2_O) δ 8.36 (s, 1H), 7.47 – 7.34 (m, 5H), 5.38 (ddd, *J* = 45.2, 17.8, 5.7 Hz, 2H), 5.23–5.10 (m, 2H), 3.59–3.44 (m, 2H), 3.18 (t, *J* = 2.6 Hz, 6H), 2.68 (dd, *J* = 6.4, 4.9 Hz, 2H). ^13^C NMR (101 mHz, D_2_O) δ 171.65, 170.08, 135.35, 128.88, 128.77, 128.45, 97.78, 95.59, 67.39, 64.87, 62.32, 48.85, 47.73, 39.95. ^19^F NMR (376 mHz, D_2_O) δ ‐192.51 (t, *J* = 8.0 Hz).

### Synthesis–Fluoromethyltosylate (FMT)

Fluoromethyl tosylate was synthesised by adapting methods reported by Smith et al.^[^ ^46^ ^]^ Methylene ditosylate (6.50 g, 1 Eq, 18.2 mmol) and caesium fluoride (9.70 g, 3.5 Eq, 63.8 mmol) were dissolved in tert‐amyl alcohol (130 mL). The reaction mixture was stirred vigorously at 90 °C for 2 h. The reaction was allowed to cool, and the tert‐amyl alcohol was removed under reduced pressure, and the slurry was extracted with ice‐cold diethyl ether. The ether was filtered and concentrated under reduced pressure to give the crude product. The crude was purified by column chromatography to give fluoromethyl 4‐methylbenzenesulfonate (1.41 g, 6.90 mmol, 37.8%) as a colourless oil. ^1^H NMR (400 mHz, CDCl_3_) δ 7.85 (d, *J* = 8.1 Hz, 2H), 7.38 (d, *J* = 8.1 Hz, 2H), 5.76 (dd, *J* = 51.0, 1.0 Hz, 2H), 2.48 (s, 3H). ^13^C NMR (101 mHz, CDCl_3_) δ 145.58, 133.88, 129.94, 127.91, 99.26, 96.96, 21.68. ^19^F NMR (376 mHz, CDCl_3_) δ ‐153.22 (t, *J* = 50.9 Hz).

### Synthesis–Fluoromethylcarnitine (FMC)

To a solution of **2** (24 mg, 1 Eq, 76 µmol) in methanol (20 mL) was added Pd/C (10 wt.%, 8.1 mg, 0.1 Eq, 7.6 µmol). The mixture was stirred under H_2_ for 18 h. The reaction mixture was then filtered through celite, and the filtrate was concentrated under reduced pressure to give FMC as a colourless gum. The gum was then triturated with CH_2_Cl_2_ to give FMC (14 mg, 76 µmol, 100%) as a white solid. ^1^H NMR (400 mHz, D_2_O) δ 5.51–5.25 (m, 2H), 4.52 (d, *J* = 6.6 Hz, 1H), 3.55–3.40 (m, 2H), 3.28–3.11 (s, 6H), 2.40 (d, *J* = 6.4 Hz, 1H). ^13^C NMR (101 mHz, D_2_O) δ 177.33, 97.80, 95.61, 65.36, 63.39, 48.88, 47.68, 42.29. ^19^F NMR (376 mHz, D_2_O) δ ‐192.79 (t, *J* = 7.8 Hz). HRMS (ESI) C_7_H_15_FNO_3_^+^ [M^+^]: calc. 180.1031, found: 180.1031

### Radiosynthesis–General Information

[^18^F]Fluoride was produced by a GE PETtrace 880 cyclotron by 16.5 MeV irradiation of an enriched [^18^O]H_2_O target, supplied by St. Thomas’ Hospital (London, UK) in ≈2.5 mL of water. [^18^F]Fluoride was used without further purification. Radioactivity was measured in a CRC‐25R dose calibrator (Capintec, Inc). Reactions were performed in 5 mL Wheaton V vials (11714239) purchased from Fisher Scientific (Loughborough, UK). Sep‐Pak QMA light cartridges (186004051) were purchased from Waters (Elstree, UK). Sep‐Pak C18 Plus and (WAT020515) Sep‐Pak C18 Plus Light Cartridges (WAT023501) were purchased from Waters (Elstree, UK) and were conditioned using EtOH (5 mL) and water (10 mL). Oasis WCX Plus Short cartridges (186003518) were purchased from Waters (Elstree, UK) and were conditioned using PBS (10 mL) and water (12 mL). Analytical RP‐HPLC was performed with an Agilent 1200 HPLC system equipped with a 1200 Series Diode Array Detector and a Raytest GABI Star NaI(Tl) scintillation detector (energy window 400–700 keV). Isolated radiochemical yield (RCY) refers to the activity of the isolated product divided by the initial activity used for the reaction. RCYs were given decay corrected. Radiochemical purity refers to the proportion of the total radioactivity in the sample which was present as the desired radiotracer, as measured by radio‐HPLC.

### Radiosynthesis–Radiosynthesis of [^18^F]fluoromethyl‐ʟ‐carnitine ([^18^F]FMC)

[^18^F]Fluoride in [^18^O]H_2_O was trapped on a QMA‐Light Sep‐Pak and then eluted into the reaction vial using aqueous K_2_CO_3_ (3.5 mg in 500 µL H_2_O). A solution of Kryptofix 222 (15 mg) in MeCN (1.0 mL) was then added to the reaction vial, and the [^18^F]fluoride/Kryptofix/carbonate mixture was dried at 110 °C under a constant flow of N_2_ for 10 min. A solution of ditosylmethane (8.0 mg) in MeCN (750 µL) and H_2_O (10 µL) was added to the vial. The vial was sealed and heated at 110 °C for 10 min. The reaction was allowed to cool for 5 min and then quenched with 15% MeCN in H_2_O (7 mL) (Note: An aliquot was taken for HPLC analysis to determine the ratio of [^18^F]fluoromethyl tosylate ([^18^F]FMT) product (retention time: 15.22 min) to [^18^F]tosylfluoride side‐product (retention time: 16.63 min)). The solution was passed dropwise through a Sep‐Pak C18 Plus Light cartridge to trap both the [^18^F]fluoromethyl tosylate and [^18^F]tosylfluoride products. The Sep‐Pak C18 Plus Light cartridge was washed with 15% MeCN in H_2_O (5 mL) and then dried by flowing N_2_ through the cartridge for 10 min (Note: A Sep‐Pak C18 Plus cartridge was attached to the exit of the C18 lite cartridge to avoid escape of radioactive volatiles or aerosols). The mixture of [^18^F]fluoromethyl tosylate and [^18^F]tosylfluoride was eluted from the C18 lite cartridge into a second V vial with precursor **1** (50 mg) in MeCN (500 µL). The reaction vial was sealed and heated at 120 °C for 45 min. The reaction was allowed to cool for 5 min and then quenched with 15% MeCN in H_2_O (7 mL) (Note: An aliquot was taken for HPLC analysis to determine the conversion of [^18^F]fluoromethyl tosylate to the benzyl protected [^18^F]fluoromethyl‐ʟ‐carnitine (**[^18^F]4**) (retention time: 11.97 min)). The solution was passed dropwise a WCX cartridge to trap the benzyl protected [^18^F]fluoromethyl‐ʟ‐carnitine (**[^18^F]4**). The WCX cartridge was then washed consecutively with aqueous NH_4_OH (5%, 10 mL), EtOH (10mL), and aqueous formic acid (2%, 10 mL). The pure benzyl protected product **[^18^F]4** was then eluted into a third reaction vial with formic acid in ethanol (1%, 4.0 mL; Note: Here, a sample (20 µL) was removed to measure the molar activity of **[^18^F]4**). The volatiles were removed at 80 °C under a constant flow of N_2_. The vial was allowed to cool for 5 min before aqueous NaOH (150 mm, 750 µL) was added to the reaction vial and the reaction was stirred at r.t. for 5 min. The reaction was neutralised with aqueous HCl (150 mm, 750 µL) and passed through a C18 lite cartridge to give pure [^18^F]fluoromethyl‐ʟ‐carnitine ([^18^F]FMC). The pH was measured and adjusted (if required) to be between pH 5.5 and 8.

### Radiosynthesis–Quality Control of [^18^F]fluoromethyl‐ʟ‐carnitine ([^18^F]FMC)

HPLC analysis of the reformulated product was conducted to assess the radiochemical purity (>95%) of the reformulated product (Eclipse XDB‐C18, 9.4 × 250 mm, 5 mm HPLC column at room temperature; solvent A: H_2_O (0.1% TFA), solvent B: MeOH (0.1% TFA); flow rate: 3.5 mL min^−1^; UV detectors: 254 and 190 nm; gradient: 5% B, 0–1 min; 5–95% B, 1–15 min; 95% B, 15–20 min, 2.0 mL injection loop, retention time = 3.32 min). The pH of the final [^18^F]FMC saline formulation was measured using Fisherbrand pH Indicator Paper Sticks (10642751).

### Radiosynthesis–Molar Activity

Molar activity of [^18^F]FMC was measured indirectly by analysis of its chromophore‐containing benzyl ester, **[^18^F]4**. The molar activity (A_M_) was calculated by measuring the UV absorbance associated with **[^18^F]4** using HPLC (see Figure S4, Supporting Information). Subsequently A_m_ (expressed in MBq µmol^−1^) was calculated using the equation:

(1) $$A_{m}=\mathrm{Activity}\;\mathrm{injected}\left(\mathrm{MBq}\right)/\mathrm{Amount}\;\mathrm{injected}\left(\mu \mathrm{mol}\right)$$

### Statistical Analysis

Statistical analysis was performed using GraphPad Prism (v.8.0). No pre‐processing was performed. All in vitro data were acquired from three or more biological replicates, acquired on separate days. All in vivo data were acquired in at least 4 animals. Data were expressed as the mean ± one standard deviation (SD). Statistical significance was determined using an unpaired two‐tailed Student's *t*‐test with Welch's correction. For analysis across multiple samples, 1‐way analysis of variance (ANOVA) followed by *t*‐tests, multiple comparison correction (Dunnet's method) was performed. Dose‐response curves were generated, and from uptake inhibition, IC_50_ values were determined using GraphPad Prism (Sigmoidal, 4PL, dose vs response (variable slope)). Differences with *p* values < 0.05 were considered statistically significant in all analyses.

## Conflict of Interest

The authors declare no conflict of interest.

## Supporting information



Supporting Information

## Data Availability

The data that support the findings of this study are available from the corresponding author upon reasonable request.
