# Supplementary material for: Fluorinated Carnitine Derivatives as Tools to Visualise Carnitine Transport and Metabolism
Source: Adv Sci (Weinh). 2025 Nov 7;13(12):e14668. doi: 10.1002/advs.202514668 (PMC12948245; doi:10.1002/advs.202514668)
Supplement: Supplementary file 1 — Supporting Information [file ADVS-13-e14668-s001.docx]

**Supporting Information**

**Fluorinated Carnitine Derivatives as Tools to Visualise Carnitine Transport and Metabolism**

Richard S. Edwards, Ella-May Hards, Sofia dos Santos, Hannah E. Greenwood, Madeleine George, Andrea Emanuelli, Muhammet Tanc, Thomas R. Eykyn and Timothy H. Witney

**Table of Contents**

1. **Supporting Figures, Schemes & Tables...…………………………………pg 2**
2. **Methods…………………………………………..………………………..…….pg 5**
3. **Synthesis……………………………………………………………..………..pg 14**
4. **Radiosynthesis………………………………………………..………………pg 19**
5. **References……………………………………………………………………..pg 22**
6. **NMR Spectra………………………………………………………………..….pg 23**

**Supporting Figures Schemes & Tables**


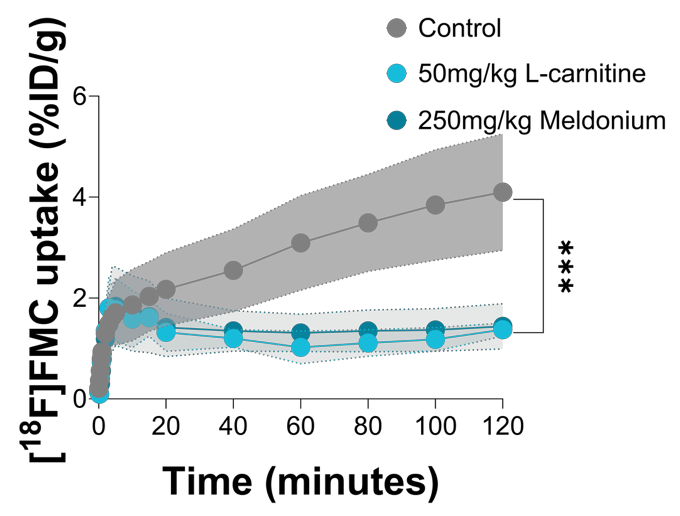


**Figure S1.** Image-derived time activity curves (TAC) for tumours from a dynamic 120-min scan following i.v. injection of [^18^F]FMC, [^18^F]FMC + carnitine and [^18^F]FMC + meldonium.

**
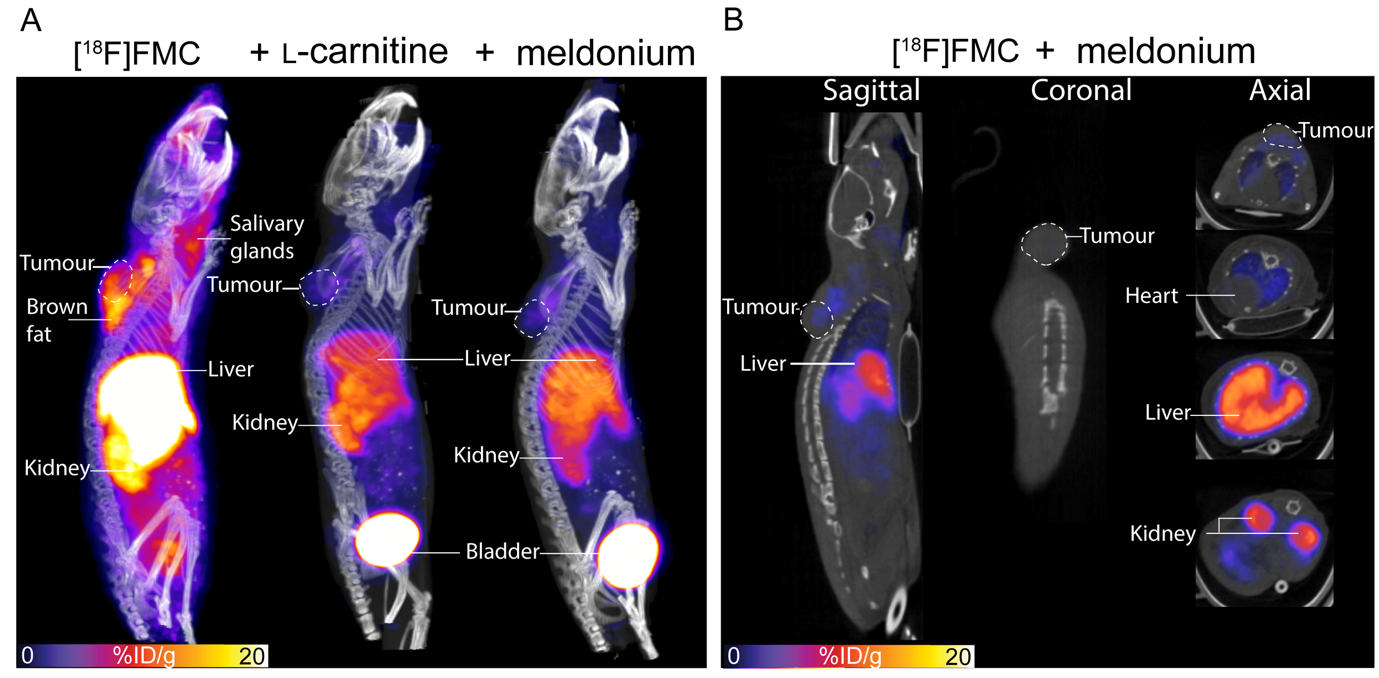
**

**Figure S2.** Tumour accumulation of [^18^F]FMC is selectively blocked by meldonium. A) Representative 100 min to 120 min maximum intensity projection PET/CT images for [^18^F]FMC (left), [^18^F]FMC + carnitine (middle) and [^18^F]FMC + meldonium (right). B) Representative 120 min sagittal, coronal and axial PET/CT images of [^18^F]FMC in a human NSCLC (H460) subcutaneous xenograft tumour model with co-injection of meldonium (250 mg/kg).

**
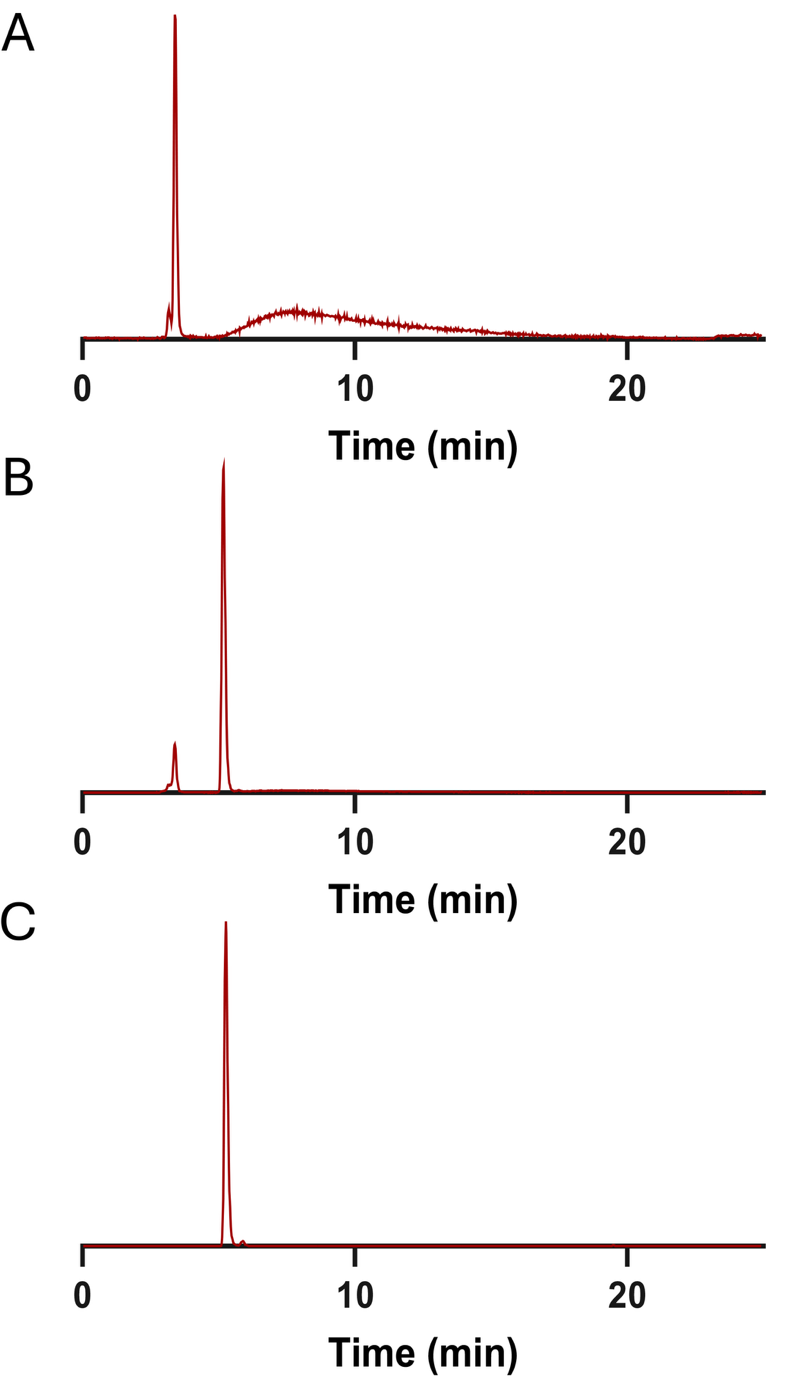
**

**Figure S3.** Radiochromatograms for [^18^F]fluoride and [^18^F]FMC. A) [^18^F]fluoride B) [^18^F]fluoride + [^18^F]FMC C) [^18^F]FMC.

**Table S1.** Full optimisation of fluoromethylation reaction.

| Entry | Solvent,  Conditions^a^ | FMT (eq) | ^19^F NMR conversion (%) | Isolated yield 2 (%) | Product purity (2:4) |
| --- | --- | --- | --- | --- | --- |
| 1 | CD_3_CN, 120 °C, 0.5 h | 1.5 | Trace | - | - |
| 2 | CD_3_CN, 120 °C, 4 h | 1.5 | 35 | - | - |
| 3^b^ | CD_3_CN, 120 °C, 4 h | 1.5 | 0 | - | - |
| 4 | CD_3_CN, 140 °C, 4 h | 1.5 | 65 | 66 | 70:30 |
| 5 | CD_3_CN, 120 °C, 4 h | 1 | 39 | 15 | 84:16 |
| 6 | DMF, 120 °C, 4 h | 1 | - | 16 | 92:8 |
| 7 | DMA, 120 °C, 4 h | 1 | - | 18 | 93:7 |
| 8 | CD_3_CN, 120 °C, 4 h | 4 | - | 25 | 98:2 |
| 9 | DMF, 120 °C, 4 h | 5 | - | 27 | 61:39 |
| 10 | DMA, 120 °C, 4 h | 2 | - | 15 | 98:2 |

^a^Reactions were performed on an 0.19-0.38 mmol scale. ^b^Norcarnitine (free carboxylate) used as starting material instead of **1**.

**Table S2.** Optimisation for [^18^F]FMT synthesis.

| Entry | Solvent,  Conditions^a^ | QMA Eluent | FMT (%) | TsF (%) | Isolated |
| --- | --- | --- | --- | --- | --- |
| 1 | MeCN (1.5% Water), 110 °C | K_222_ (15 mg), K_2_CO_3_ (3.5 mg) | 63 | 37 |  |
| 2 | MeCN (10% EtOH), 110 °C | K_222_ (8 mg), K_2_CO_3_ (1.1 mg) | 52 | 48 |  |
| 3 | MeCN (10% EtOH), 110 °C | TEABC (4.0 mg) | 63 | 37 |  |
| 3 | MeCN (10% EtOH), 85 °C | TEABC (4.0 mg) | 59 | 41 |  |
| 5 | MeCN (1.5% Water), 110 °C | K_222_ (15 mg), K_2_CO_3_ (3.5 mg) | 69 | 31 | 85 |
| 6 | Isobutanol/MeCN (5:1), 110 °C | K_222_ (15 mg), K_2_CO_3_ (3.5 mg) | 72 | 28 | 33 |

^a^ 10 min reaction time

**Table S3.** Percentage of [^18^F]FMC and corresponding metabolites in tissue samples, as determined by radio-HPLC.

| Tissue | [^18^F]FMC (%) | [^18^F]Acetyl-FMC (%) | [^18^F]Acyl-FMCs (%) |
| --- | --- | --- | --- |
| Tumour | 67.8 ± 1.8 | 28.3 ± 3.7 | 4.0 ± 3.1 |
| Liver | 72.1 ± 2.6 | 23.4 ± 1.5 | 4.6 ± 1.3 |
| Kidney | 71.5 ± 3.2 | 23.9 ± 2.7 | 4.7 ± 0.8 |
| Heart | 68.0 ± 10.7 | 25.2 ± 9.0 | 7.2 ± 1.6 |
| Blood | 45.5 ± 2.7 | 52.7 ± 4.7 | 2.1 ± 1.9 |

**Methods**

**Cell culture**

Human NSCLC H460 cells were maintained in RPMI 1640 media (ThermoFisher Scientific) supplemented with 10% foetal bovine serum (FBS) (ThermoFisher Scientific) and 100 U/mL penicillin, 100 μg/mL streptomycin (Sigma Aldrich Ltd.). Cells were maintained in a humidified atmosphere at 37 °C and 5% CO_2_.

Cells were seeded for 24 h prior to experimental endpoint in 6-well plates in 2mL of RPMI 1640(Thermo Fisher). All cells were seeded at 1.5 × 10^5^/mL. For siRNA experiments control cells were seeded at 1.5 × 10^5^/mL and control siRNA and OCTN2 siRNA treated cells were seeded at 1.75 × 10^5^/mL

**Quantitative PCR**

Total RNA was isolated from collected cell pellets using RNeasy mini kit (Qiagen, Cat:74104) and reverse transcribed using the SuperScript IV Master Mix (Thermofisher, Cat: 11756050). Quantitative real-time PCR was performed on a Quantstudio 3 (Thermofisher) using the following Gene Expression Assays on Demand (ThermoFisher Scientific; 4331182): OCTN2/SLC22A5, Hs00929869_m1; CPT1a, Hs00912671_m1 and TATA-box binding protein (TBP) endogenous control, (ThermoFisher Scientific; 4448489), Hs00427620_m1. Target gene expression was normalized to a Universal RNA (QS0639) reference sample using the ΔΔCt method. For siRNA experiments target gene expression was normalised to control samples.

**OCTN2 silencing using RNAi**

OCTN2 protein expression was modulated in culture through small inhibitory RNA (ON-TARGETplus siRNA, Horizon discovery). H460 NSCLC cells were seeded into 6-well plates 24 h prior to transfection with OCTN2 or control siRNA (L-007456-00-0005/D-001920-01-05, Horizon discovery). 25 nM of siRNA in 200 µL per well of Optimem (ThermoFisher) was prepared in tube A whilst 200 µL per well of transfection reagent (T-2001-03, Horizon discovery) was prepared in tube B. Tube A and B were mixed and incubated for 5 minutes. The siRNA and transfection mixture was added to cells and topped up with 1600 μL of antibiotic-free complete medium (RPMI: Thermofisher) for a total volume of 2000 μL per well and incubated for 8 h. The transfection mixture was then replaced with normal antibiotic-free complete medium (RPMI: Thermofisher) for 72 h.

**Uptake and blocking**

0.185 MBq/mL solutions of radiotracer in fresh Hanks' Balanced Salt Solution (HBSS) prewarmed to 37 °C were prepared. Media was replaced with the radioactivity containing media. Plates were then incubated for 60 min at 37 °C and 5 % CO_2_.

For competition studies, inhibitors (meldonium) and transporter substrates (γ-butytrobetaine, L-carnitine, palmitoylcarnitine) were co-incubated with [^18^F]FMC at a range of concentrations (1000 μM-0.1 μM) in 6 well plates for 60 min at 37°C and 5 % CO_2_. Uptake is expressed as % vehicle treated control.

Following the appropriate incubation time, plates were placed on ice and washed three times with ice-cold PBS to remove exogenous radioactivity. RIPA buffer (500 μL in 6 well plates; Fisher Scientific Ltd) was added to each well to lyse the cells, and cells were scraped for efficient cell lysis. Decay-corrected radioactivity was determined on a gamma counter (300 μL of lysate; 2480 WIZARD2 automated gamma counter, PerkinElmer) and the remaining cell lysate was used to determine protein concentration following radioactive decay (Pierce BCA assay). To quantify radiotracer uptake, three 10 μL standard solutions of the radioactivity-containing media were counted on the gamma counter, accounting for 1% of the added dose. Counts acquired from the gamma counter were adjusted to account for the whole 500 μL of cell lysate. Data were expressed as a percent of total radioactivity added to cells per mg of protein.

**^19^F NMR of cell lysates**

^19^F-FMC at a concentration of 50 μM in RPMI was incubated with H460 cells for 24 h before harvesting. 1 mL of culture media was removed for later analysis and stored at -20 °C. Fresh samples of medium were also collected. Next, 3 mL of ice-cold deuterated methanol (Merck) was added to each flask and kept on ice for 5-10 minutes. Cells were harvested by scraping the surface, and the cell/methanol suspensions were centrifuged for 5 minutes at 12,000 × *g* at 4 °C. The supernatant was removed and concentrated to a final volume of 540 μL in deuterated methanol. 60 μL of D_2_O buffer was added to each sample before NMR spectral acquisition. A standard of ^19^F-FMC at a concentration of 50 μM was prepared in deuterated methanol. NMR work was performed at the UCL School of pharmacy by Dr Nikita Harvey. ^19^F nuclear magnetic resonance spectra were acquired using a vertical-bore, ultra-shielded Bruker (Karlsruhe, Germany) 14.1 T (600 MHz) spectrometer equipped with a QCI-F cryoprobe, at 298 K using the Bruker zgig pulse program for ^1^H decoupling. Acquisition parameters were: 1024 scans; 4 dummy scans; 20.1 ppm spectral width; acquisition time 0.36 s; and pre-scan delay 0.5 s. TopSpin (version 4.0.5) software was used for data acquisition and for metabolite quantification. Free induction decays (FIDs) were multiplied by a line broadening factor of 0.5 Hz and Fourier transformed, phase, and automatic baseline corrected.

**Mass spectrometry**

The samples prepared for ^19^F NMR analysis (see above) were fractionated by HPLC (25×1 min fractions). Eclipse XDB-C18, 9.4 × 250 mm, 5 mm HPLC column at room temperature; solvent A: H_2_O (0.1% TFA), solvent B: MeOH (0.1% TFA); flow rate: 3.5 mL/min; UV detectors: 254 nm and 190 nm; gradient: 0% B, 0–3 min; 0–5% B, 3–11 min; 5–95% B, 11–20 min; 95-0% B, 20–25 min, 5.0 mL injection loop. The samples were analysed using a Waters G2-XS QTof with a Waters Acquity I class UPLC. Waters ACQUITY UPLC BEH C18, 1.7 µm, 2.1 mm x 50 mm HPLC column 60 °C; solvent A: H_2_O (0.1% FA), solvent B: MeCN (0.1% FA); flow rate: 0.4 mL/min; +ve ionisation mode; gradient: 2% B, 0–1 min; 2–75% B, 1–4.5 min; 75–95% B, 4.5–4.6 min; 95% B, 4.6–5.6 min; 95–2% B, 5.6–5.7 min; 2% B, 5.7–6.5 min, Capillary: 3.0 kV, Sampling Cone: 40.0, Source Temperature: 120 °C, Desolvation Temperature: 250 °C, Desolvation Gas Flow: 600.0 L/Hr.

**Cell metabolism with radio-HPLC**

H460 cells were seeded in 10 cm^3^ cell culture dish 24 h prior to experiment. 0.5 MBq/mL solutions of radiotracer in 5mL of fresh Hanks' Balanced Salt Solution (HBSS) prewarmed to 37 ^o^C were prepared. Media was replaced with the radioactivity containing media. Plates were then incubated for 15, 30 and 60 min at 37 °C and 5% CO_2_ before being place on ice and the exogenous radioactivity removed. The cells were washed three times with ice cold PBS (3×5 mL) and then lysed with MeOH (5 mL) and scrapped. The supernatant was transferred to a glass vial through a Millex 0.2 µm filter (Millipore, Billerica, MA, USA). The samples were evaporated at room temperature using an Asynt smart evaporator, diluted with 2 mL mobile phase (H_2_O, 0.1% TFA), passed through another Millex 0.2 µm filter (Millipore, Billerica, MA, USA) and monitored by reverse phase HPLC. Eclipse XDB-C18, 9.4 × 250 mm, 5 mm HPLC column at room temperature; solvent A: H_2_O (0.1% TFA), solvent B: MeOH (0.1% TFA); flow rate: 3.5 mL/min; UV detectors: 254 nm and 190 nm; gradient: 0% B, 0–3 min; 0–5% B, 3–11 min; 5–95% B, 11–20 min; 95-0% B, 20–25 min, 2.0 mL injection loop).

**Animal studies**

All animal experiments were performed in accordance with the United Kingdom Home Office Animal (scientific procedures) Act 1986. PPL licence used was number I27111203.

**Tumour models**

For imaging, 3 × 10^6^ H460 cancer cells in 100 µL PBS were injected subcutaneously into female Balb/C nu/nu mice aged 6-9 weeks (Charles River Laboratories). Tumour growth was monitored using an electronic calliper and the volume calculated using the following equation: volume = ((π/6) × h × w × l), where h, w and l represent, height, width and length, respectively. Tumour size was monitored daily, with studies taking place when tumour volume reached ~100 mm^3^.

***In vivo* imaging**

For all imaging studies, mice were maintained under anaesthesia with isoflurane (1.5-2% in O2) at 37 °C during tail vein cannulation and imaging. For healthy imaging, dynamic PET scans were acquired on a Mediso NanoScan PET/CT system (1-5 coincidence mode; 3D reconstruction; CT attenuation-corrected; scatter corrected) using the four-bed mouse hotel(39). Images were acquired for 120 min following a bolus intravenous injection of [^18^F]FMC (~ 1.5-3 MBq in 100 μL) through a tail vein cannula. For the LC co-injection study, 400 μM of LC (Sigma) was simultaneously injected in 50 uL through a tail vein cannula. To determine radiotracer specificity in H460 tumour models, LC (50 mg/kg; *n*=4/group) and meldonium (250 mg/kg; *n*=4/group) were co-injected i.v. with [^18^F]FMC. CT images were obtained for anatomical reference and attenuation correction (180 projections; semicircular acquisition; 50 kVp; 300 ms exposure time). The acquired data was reconstructed into 15 bins of 4 × 15 seconds, 4 × 60 seconds, and 3 × 300 seconds, 4 x 20 minutes (Tera-Tomo 3D reconstructed algorithm; 4 iterations; 6 subjects; 400-600 keV; 0.3 mm^3^ voxel size). VivoQuant software (v 2.5, Invicro Ltd.) was used to analyse the reconstructed images. Regions of interest (ROIs) were drawn manually using CT images and 120-minute dynamic PET images. Time verses radioactivity curves (TACs) were generated using the percentage injected dose per mL (%ID/g).

**Biodistribution**

Approximately ~1.5 MBq of radiotracer was injected via the tail vein of conscious healthy mice (*n* **=** 4 per group). After 120 min, animals were sacrificed, and organs and tissues of interest were collected and weighed. The amount of radioactivity in each tissue was determined with the gamma counter to calculate uptake as % injected dose per g of wet weight tissue (ID/g).

**In vivo metabolite analysis**

In vivo metabolism of [^18^F]FMC was performed by radio-HPLC analysis. Metabolites and parent tracer were quantified based on the area under the curve (region of interest) for [^18^F]FMC and its corresponding metabolites ([^18^F]Acetyl-FMC and [^18^F]Acyl-FMCs) and expressed as a percentage (mean ± SD). Tumour, heart, blood, liver, and kidney samples were analysed at 120 min post-injection of the tracer.

H460 cells (3 × 10^6^) in PBS were injected into the flank of female Balb/c nu/nu mice. When the tumors reached ~100 mm^3^ mice were anesthetized with isoflurane (1.5-2% in oxygen) and injected with ~5 MBq of radiotracer through a tail vein cannular. Mice were maintained at 37°C under anesthesia throughout radiotracer uptake. At 2 h p.i., the mice were sacrificed by exsanguination via cardiac puncture under terminal anaesthesia. Tumour, heart, blood, liver, and kidney samples were harvested and placed on ice prior to processing. The blood samples were centrifuged (2,000 × g for 5 min, 4°C), the plasma was removed and transferred to a 1.5 mL Eppendorf. Ice-cold MeOH (1 mL) was added to the plasma and the sample was briefly mixed on a Vortex. On ice, ice-cold MeOH (1 mL) was added to the heart, liver, tumour and kidney samples prior to homogenization using a PRECELLYS® 24 tissue homogenizer. All samples were then centrifuged (12,000 × g for 5 min, 4°C) and the supernatant transferred to a glass vial through a Millex 0.2 µm filter (Millipore, Billerica, MA, USA). The samples were evaporated at room temperature using an Asynt smart evaporator, diluted with 2 mL mobile phase (H_2_O, 0.1% TFA), passed through another Millex 0.2 µm filter (Millipore, Billerica, MA, USA) and monitored by reverse phase HPLC. Eclipse XDB-C18, 9.4 × 250 mm, 5 mm HPLC column at room temperature; solvent A: H_2_O (0.1% TFA), solvent B: MeOH (0.1% TFA); flow rate: 3.5 mL/min; UV detectors: 254 nm and 190 nm; gradient: 0% B, 0–3 min; 0–5% B, 3–11 min; 5–95% B, 11–20 min; 95-0% B, 20–25 min, 2.0 mL injection loop, retention time [^18^F]FMC = 5.55 min).

**Statistics**

Statistical analysis was performed using GraphPad Prism (v.8.0). All *in vitro* data was acquired from three or more biological replicates, acquired on separate days. Data were expressed as the mean ± one standard deviation (SD). Statistical significance was determined using unpaired two-tailed Student’s t-test with Welch’s correction. For analysis across multiple samples, 1-way analysis of variance (ANOVA) followed by t-tests multiple comparison correction (Dunnet’s method) were performed. Dose-response curves were generated, and from uptake inhibition, IC_50_ values were determined using GraphPad Prism (v.8.0; (Sigmoidal, 4PL, dose vs response (variable slope)). Differences with p values < 0.05 were considered statistically significant in all analyses.

**Synthesis**

**General Information**

Commercially available starting materials were purchased from Sigma-Aldrich, Alfa Aesar and Apollo Scientific and were used without further purification. Norcarnitine was purchased from BioServUK. Solvents were obtained from Sigma-Aldrich; unless stated otherwise, reagent grade solvents were used for reactions and column chromatography. Unless otherwise specified, ‘water’ refers to sterile ultrapure water (18.2 MΩ-cm). Reaction progress was monitored by thin layer chromatography (TLC) on aluminium sheets coated with silica gel 60 F254 (Merck Millipore) and detection was carried out using UV light (325 nm and 254 nm) and/or chemical solutions. Crude reaction mixtures were purified by automated flash column chromatography (Biotage Isolera One). Microwave reactions were performed using a CEM Discovery SP microwave synthesiser. ^1^H, ^13^C, and ^19^F Nuclear Magnetic Resonance (NMR) spectra were recorded on a Bruker Avance 400 equipped with a BBFO probe at room temperature. ^13^C NMR experiments were proton decoupled. ^1^H and ^13^C NMR spectra are reported relative to the internal reference of the relative deuterated solvent. Chemical shifts (d) are reported in ppm and coupling constants (J) are given in Hertz (Hz). Multiplicity is described with (s): singlet, (d): doublet, (t): triplet and (q): quadruplet. High resolution mass spectrometry data were recorded on a Waters G2-XS QTof with a Waters Acquity I class UPLC. Waters ACQUITY UPLC BEH C18, 1.7 µm, 2.1 mm x 50 mm HPLC column 60 °C; solvent A: H_2_O (0.1% FA), solvent B: MeCN (0.1% FA); flow rate: 0.4 mL/min; +ve ionisation mode; gradient: 2% B, 0–1 min; 2–75% B, 1–4.5 min; 75–95% B, 4.5–4.6 min; 95% B, 4.6–5.6 min; 95–2% B, 5.6–5.7 min; 2% B, 5.7–6.5 min, Capillary: 3.0 kV, Sampling Cone: 40.0, Source Temperature: 120 °C, Desolvation Temperature: 250 °C, Desolvation Gas Flow: 600.0 L/Hr.

**Benzyl (*R*)-4-(dimethylamino)-3-hydroxybutanoate (1)**

(*R*)-4-(dimethylamino)-3-hydroxybutanoic acid (norcarnitine; 2.0 g, 1.0 Eq, 13.6 mmol) was dissolved in benzyl alcohol (50.0 ml) to give a colourless suspension. Thionyl chloride (1.94 g, 1.19 mL, 1.2 Eq, 16.3 mmol) was added dropwise. The reaction was stirred at 70 °C for 2 h under N_2_. The solution was then allowed to cool to room temperature and the volatiles were removed under reduced pressure. The residue was treated with 50 ml of HCl (0.5 N) and washed with ether (3×30 mL). Saturated sodium bicarbonate solution was added to the aqueous layer to adjust the pH to 8.0–8.5. The aqueous solution was then continuously extracted with dichloromethane (100 mL) for 2 h. The organic layer was dried with anhydrous Na_2_SO_4_ and concentrated to give benzyl (*R*)-4-(dimethylamino)-3-hydroxybutanoate (**1**) (2.59 g, 10.91 mmol, 80.3 %) as a colourless oil.

^1^H NMR (400 MHz, CDCl_3_) δ 7.43 – 7.23 (m, 5H), 5.17 (s, 2H), 4.21 – 4.06 (m, 1H), 2.59 – 2.44 (m, 2H), 2.42 – 2.20 (m, 2H), 2.28 (s, 6H). ^13^C NMR (101 MHz, CDCl_3_) δ 171.55, 135.85, 128.57, 128.25, 128.24, 66.40, 64.64, 64.39, 45.59, 39.84.

**(*R*)-4-(benzyloxy)-*N*-(fluoromethyl)-2-hydroxy-N,N-dimethyl-4-oxobutan-1-aminium formate (2)**

A mixture of benzyl (*R*)-4-(dimethylamino)-3-hydroxybutanoate (**1**) (45 mg, 1.0 Eq, 190 µmol) and FMT (194 mg, 5.0 Eq, 950 µmol) in CD_3_CN (2.0 mL) was added to a microwave reaction tube (2.0 – 5.0 mL). The mixture was then heated by microwave irradiation at 120 °C for 1 h. The reaction mixture was allowed to cool to room temperature before being diluted with water (20 mL) and CH_2_Cl_2_ (20 mL). The two layers were mixed thoroughly and partitioned using a separating funnel. The aqueous layer was divided in two and each portion was passed through a WCX cartridge (preconditioned with 5% aqueous NH_4_OH). The WCX cartridges were then washed consecutively with aqueous NH_4_OH (5%, 10 mL), EtOH (10mL) and aqueous formic acid (1%, 10 mL). The product **2** was eluted into a round bottomed flask with a solution of formic acid in ethanol (2%, 10 mL). The volatiles were removed to give **2** as it’s formic acid salt (15 mg, 190 µmol, 25%) as a colourless oil.

^1^H NMR (400 MHz, D_2_O) δ 8.36 (s, 1H), 7.47 – 7.34 (m, 5H), 5.38 (ddd, *J* = 45.2, 17.8, 5.7 Hz, 2H), 5.23 – 5.10 (m, 2H), 3.59 – 3.44 (m, 2H), 3.18 (t, *J* = 2.6 Hz, 6H), 2.68 (dd, *J* = 6.4, 4.9 Hz, 2H). ^13^C NMR (101 MHz, D_2_O) δ 171.65, 170.08, 135.35, 128.88, 128.77, 128.45, 97.78, 95.59, 67.39, 64.87, 62.32, 48.85, 47.73, 39.95. ^19^F NMR (376 MHz, D_2_O) δ -192.51 (t, *J* = 8.0 Hz).

**Ditosylmethane (3)**

Diiodomethane (6.00 g, 1.81 mL, 1.0 Eq, 22.4 mmol) was added to Silver toluene-4-sulphonate (13.8 g, 2.2 Eq, 49.3 mmol) in anhydrous acetonitrile (100 mL) and the mixture was heated at reflux for 18 h. The mixture was cooled to room temperature and the solvent was removed under reduced pressure. The resultant residue was extracted with DCM at 40 °C and filtered through celite. The filtrate was concentrated to give methylene bis(4-methylbenzenesulfonate) (6.5 g, 18 mmol, 81 %) as a white solid.

^1^H NMR (400 MHz, CDCl_3_) δ 7.51 (d, *J* = 8.4 Hz, 4H), 7.17 (d, *J* = 7.9 Hz, 4H), 5.74 (s, 2H), 2.37 (s, 6H).

**Fluoromethyltosylate (FMT)**

Fluoromethyl tosylate (**FMT**) was synthesised by adapting methods reported by Smith *et al.*^[1]^ Methylene ditosylate (6.50 g, 1 Eq, 18.2 mmol) and caesium fluoride (9.70 g, 3.5 Eq, 63.8 mmol) were dissolved in tert-amyl alcohol (130 mL). The reaction mixture was stirred vigorously at 90 °C for 2 h. The reaction was allowed to cool, and the tert-amyl alcohol was removed under reduced pressure and the slurry was extracted with ice-cold diethyl ether. The ether was filtered and concentrated under reduced pressure to give the crude product. The crude was purified by column chromatography to give fluoromethyl 4-methylbenzenesulfonate (1.41 g, 6.90 mmol, 37.8%) as a colourless oil.

^1^H NMR (400 MHz, CDCl_3_) δ 7.85 (d, *J* = 8.1 Hz, 2H), 7.38 (d, *J* = 8.1 Hz, 2H), 5.76 (dd, *J* = 51.0, 1.0 Hz, 2H), 2.48 (s, 3H). ^13^C NMR (101 MHz, CDCl_3_) δ 145.58, 133.88, 129.94, 127.91, 99.26, 96.96, 21.68. ^19^F NMR (376 MHz, CDCl_3_) δ -153.22 (t, *J* = 50.9 Hz).

**Fluoromethylcarnitine (FMC)**

To a solution of **2** (24 mg, 1 Eq, 76 µmol) in methanol (20 mL) was added Pd/C (10 wt.%, 8.1 mg, 0.1 Eq, 7.6 µmol). The mixture was stirred under H_2_ for 18 h. The reaction mixture was then filtered through celite, and the filtrate was concentrated under reduced pressure to give FMC as a colourless gum. The gum was then triturated with CH_2_Cl_2_ to give FMC (14 mg, 76 µmol, 100%) as a white solid.

^1^H NMR (400 MHz, D_2_O) δ 5.51 – 5.25 (m, 2H), 4.52 (d, *J* = 6.6 Hz, 1H), 3.55 – 3.40 (m, 2H), 3.28 – 3.11 (s, 6H), 2.40 (d, *J* = 6.4 Hz, 1H). ^13^C NMR (101 MHz, D_2_O) δ 177.33, 97.80, 95.61, 65.36, 63.39, 48.88, 47.68, 42.29. ^19^F NMR (376 MHz, D_2_O) δ -192.79 (t, *J* = 7.8 Hz). HRMS (ESI) C_7_H_15_FNO_3_^+^ [M^+^]: calc. 180.1031, found: 180.1031

**Radiosynthesis**

**General Information**

[^18^F]Fluoride was produced by a GE PETtrace 880 cyclotron by 16.5 MeV irradiation of enriched [^18^O]H_2_O target, supplied by St. Thomas’ Hospital (London, UK) in approximately 2.5 mL of water. [^18^F]Fluoride was used without further purification. Radioactivity was measured in a CRC-25R dose calibrator (Capintec, Inc). Reactions were performed in 5 mL Wheaton® V vials (11714239) purchased from Fisher Scientific (Loughborough, UK). Sep-Pak® QMA light cartridges (186004051) were purchased from Waters (Elstree, UK). Sep-Pak® C18 Plus and (WAT020515) Sep-Pak® C18 Plus Light Cartridges (WAT023501) were purchased from Waters (Elstree, UK) and were conditioned using EtOH (5 mL) and water (10 mL). Oasis® WCX Plus Short cartridges (186003518) were purchased from Waters (Elstree, UK) and were conditioned using PBS (10 mL) and water (12 mL). Analytical RP-HPLC was performed with an Agilent 1200 HPLC system equipped with a 1200 Series Diode Array Detector and a Raytest GABI Star NaI(Tl) scintillation detector (energy window 400–700 keV). Isolated radiochemical yield (RCY) refers to the activity of the isolated product divided by the initial activity used for the reaction. RCYs are given decay corrected. Radiochemical purity refers to the proportion of the total radioactivity in the sample which is present as the desired radiotracer, as measured by radio-HPLC.

**Radiosynthesis of [^18^F]fluoromethyl-ʟ-carnitine ([^18^F]FMC)**

[^18^F]Fluoride in [^18^O]H_2_O was trapped on a QMA-Light Sep-Pak and then eluted into the reaction vial using aqueous K_2_CO_3_ (3.5 mg in 500 µL H_2_O). A solution of Kryptofix® 222 (15 mg) in MeCN (1.0 mL) was then added to the reaction vial and the [^18^F]fluoride/Kryptofix/carbonate mixture was dried at 110 °C under a constant flow of N_2_ for 10 min. A solution of ditosylmethane (8.0 mg) in MeCN (750 µl) and H_2_O (10 µl) was added to vial. The vial was sealed and heated at 110 °C for 10 min. The reaction was allowed to cool 5 min and then quenched with 15% MeCN in H_2_O (7 mL) (Note: An aliquot was taken for HPLC analysis to determine the ratio of [^18^F]fluoromethyl tosylate ([^18^F]FMT) product (retention time: 15.22 min) to [^18^F]tosylfluoride side-product (retention time: 16.63 min)). The solution was passed dropwise through a Sep-Pak® C18 Plus Light cartridge to trapping both the [^18^F]fluoromethyl tosylate and [^18^F]tosylfluoride products. The Sep-Pak® C18 Plus Light cartridge was washed with 15% MeCN in H_2_O (5 mL) and then dried by flowing N_2_ through the cartridge for 10 min (Note: A Sep-Pak® C18 Plus cartridge was attached to the exit of the C18 lite cartridge to avoid escape of radioactive volatiles or aerosols). The mixture of [^18^F]fluoromethyl tosylate and [^18^F]tosylfluoride was eluted from the C18 lite cartridge into a second V vial with precursor **1** (50 mg) in MeCN (500 µl). The reaction vial was sealed and heated at 120 °C for 45 min. The reaction was allowed to cool 5 min and then quenched with 15% MeCN in H_2_O (7 mL) (Note: An aliquot was taken for HPLC analysis to determine the conversion of [^18^F]fluoromethyl tosylate to the benzyl protected [^18^F]fluoromethyl-ʟ-carnitine (**[^18^F]4**) (retention time: 11.97 min)). The solution was passed dropwise a WCX cartridge to trap the benzyl protected [^18^F]fluoromethyl-ʟ-carnitine (**[^18^F]4**). The WCX cartridge was then washed consecutively with aqueous NH_4_OH (5%, 10 mL), EtOH (10mL) and aqueous formic acid (2%, 10 mL). The pure benzyl protected product **[^18^F]4** was then eluted into a third reaction vial with formic acid in ethanol (1%, 4.0 mL; *Note: Here, a sample (20 µL) was removed to measure the molar activity of* ***[^18^F]4***). The volatiles were removed at 80 °C under a constant flow of N_2_. The vial was allowed to cool for 5 min before aqueous NaOH (150 mM, 750 µL) was added to the reaction vial and the reaction was stirred at r.t. for 5 min. The reaction was neutralised with aqueous HCl (150 mM, 750 µL) and passed through a C18 lite cartridge to give pure [^18^F]fluoromethyl-ʟ-carnitine ([^18^F]FMC). The pH was measured and adjusted (if required) to be between pH 5.5 and 8.

**Quality Control of [^18^F]fluoromethyl-ʟ-carnitine ([^18^F]FMC)**

HPLC analysis of the reformulated product was conducted to assess the radiochemical purity (>95%) of the reformulated product (Eclipse XDB-C18, 9.4 × 250 mm, 5 mm HPLC column at room temperature; solvent A: H_2_O (0.1% TFA), solvent B: MeOH (0.1% TFA); flow rate: 3.5 mL/min; UV detectors: 254 nm and 190 nm; gradient: 5% B, 0–1 min; 5–95% B, 1–15 min; 95% B, 15–20 min, 2.0 mL injection loop, retention time = 3.32 min). The pH of the final [^18^F]FMC saline formulation was measured using Fisherbrand™ pH Indicator Paper Sticks (10642751).

**Molar Activity**

Molar activity of [^18^F]FMC was measured indirectly by analysis of its chromophore containing benzyl ester, **[^18^F]4**. The molar activity (A_M_) was calculated by measuring the UV absorbance associated with **[^18^F]4** using HPLC (see Supplemental Figure S4). Subsequently A_M_ (expressed in MBq/μmol) was calculated using the equation:

A_M_ = Activity injected (MBq)/ Amount injected (μmol)

**Figure S4.** HPLC UV calibration curve for **[^18^F]4**.

**References**

[1] K. L. Brocklesby, J. S. Waby, C. Cawthorne, G. Smith, *Tetrahedron Lett* **2018**, *59*, 1635-1637.

**^1^H NMR - Benzyl (*R*)-4-(dimethylamino)-3-hydroxybutanoate (1)**

**^13^C NMR - Benzyl (*R*)-4-(dimethylamino)-3-hydroxybutanoate (1)**

**^1^H NMR - (*R*)-4-(benzyloxy)-*N*-(fluoromethyl)-2-hydroxy-N,N-dimethyl-4-oxobutan-1-aminium formate (2)**

**^1^H NMR with solvent suppression (*R*)-4-(benzyloxy)-*N*-(fluoromethyl)-2-hydroxy-N,N-dimethyl-4-oxobutan-1-aminium formate (2)**

**^13^C NMR - (*R*)-4-(benzyloxy)-*N*-(fluoromethyl)-2-hydroxy-N,N-dimethyl-4-oxobutan-1-aminium formate (2)******

**^1^H NMR – Ditosylmethane (3)**

**^1^H NMR - Fluoromethyltosylate (FMT)**

**^13^C NMR - Fluoromethyltosylate (FMT)**

**^1^H NMR – Fluoromethylcarnitine (FMC)**

**^1^H NMR with solvent suppression – Fluoromethylcarnitine (FMC)**

**^13^C NMR – Fluoromethylcarnitine (FMC)**
